# The *Solanum chacoense Fertilization-Related Kinase 3 (ScFRK3)* is involved in male and female gametophyte development

**DOI:** 10.1186/s12870-019-1804-0

**Published:** 2019-05-16

**Authors:** Caroline Daigle, Benjamin Mazin, Daniel P. Matton

**Affiliations:** Institut de recherche en biologie végétale, Département de sciences biologiques, Université de Montréal, 4101 rue Sherbrooke Est, Montréal, QC Canada

**Keywords:** Mitogen-activated protein kinase kinase kinase (MAPKKK or MEKK), Mitogen-activated protein kinase kinase (MAPKK or MEK), Mitogen-activated protein kinase (MAPK or MPK), Signaling cascade, Reproduction, Gametophyte, Embryo sac, Pollen, *Solanum chacoense*

## Abstract

**Background:**

The Fertilization-related kinases (FRK) form a class that belongs to the MEKK subfamily of plant MAPKKKs. It was recently shown that FRK class kinases expanded during angiosperm evolution, reaching their maximum numbers in the lineage leading to solanaceous species and culminating in the Solanum genus where they account for more than 40% of the total MEKKs. The first members studied, *ScFRK1* and *ScFRK2* were shown to play a pivotal role in gametophyte development in the wild potato species *Solanum chacoense.*

**Results:**

*ScFRK3* is also involved in gametophyte development. *ScFRK3* is expressed in developing pollen and young ovules, reaching its highest level immediately after meiosis and during the mitosis steps in both gametophytes. Hence, three independent lines of *ScFRK3* RNAi mutant plants showed decreased number of seeds per fruit. We also observed an important number of degenerated embryo sac in mature ovary. Analysis of ovule development showed that most embryo sac did not enter mitosis I in Sc*FRK3* RNAi mutant plants. Severe lethality was also observed during male gametophyte development, pollen being arrested before mitosis I, as observed in the female gametophyte. Obvious defects in vegetative organs were not observed, emphasizing the reproductive roles of the FRK class kinases. To isolate MAP kinases acting downstream of ScFRK3, a de novo *S. chacoense* transcriptome from male and female reproductive organs was assembled. Of the five ScMKKs and 16 ScMPKs retrieved, only the ScMKK3 interacted with ScFRK3, while only the ScMPK13 interacted with ScMKK3, leading to an apparent single three-tiered canonical MAP kinase cascade combination involving ScFRK3-ScMKK3-ScMPK13.

**Conclusions:**

The ScFRK3 MAPKKK is involved in a signaling cascade that regulates both male and female gamete development, and most probably act upstream of ScMKK3 and ScMPK13.

**Electronic supplementary material:**

The online version of this article (10.1186/s12870-019-1804-0) contains supplementary material, which is available to authorized users.

## Background

Unlike in animals, gametes development in plants is a highly regulated process resulting not only from a meiosis, but also by several mitosis, giving rise to multicellular gametophytes. In most species, the female gametophyte, which is called the embryo sac (ES), comprises at maturity eight nuclei embedded in seven cells [1]. Development of the female gametophyte is divided in two steps, megasporogenesis and megagametogenesis. In megasporogenesis, the megaspore mother cell (MMC) undergoes through meiosis, resulting into four haploid tetrads of which only one will survive to become the functional megaspore (FM). During megagametogenesis, the female gametophyte (FG) undergoes three successive mitosis rounds. The resulting eight nuclei ES then becomes polarized, with four nuclei at the chalazal pole and four at the micropylar pole. One nuclei from each pole migrate to the center while others go through cellularization and differentiation. At the mature stage (FG7), two synergids and one egg cell are positioned at the micropylar pole while the three other cells, the antipodals, occupy the chalazal pole. The two nuclei in the center merge together to become the unique diploid central cell [1, 2]. On the male side, the pollen mother cell (PMC) undergoes two rounds of meiosis to produce a tetrad of microspores. The first pollen mitosis (PMI) occurs after maturation of the haploid microspore, producing two different cells inside the pollen grain: a large central cell, called the vegetative cell, and a smaller one, the generative cell [3]. Once pollen grains land on the stigma of a suitable flower of the same species, the vegetative cell generates a pollen tube that will elongate through the style toward the ovules. In solanaceous species like *Solanum chacoense*, the second pollen mitosis (PMII) occurs within the generative cell during tube elongation to form two identical sperm cells [3]. When the pollen tube reaches the micropyle of the ovule, the two sperm cells are released from the pollen tube to fertilize the egg cell and the central cell, leading to double fertilization giving rise to the diploid embryo and the triploid endosperm [4].

Cell-cell communication and coordination of male and female gametophyte development involves complex and tightly regulated steps. Accordingly, genes shown to be essential for gametophyte development are found in a wide array of functionalities [1]. Some of them are necessary for the megaspore mother cell (MMC) meiosis [5], DNA recombination and chromosome integrity [6], while others are involved in FG maintenance and mitosis, their mutation leading to developmental arrest of the embryo sac before mitosis I [7, 8]. Because of its simple structure, pollen has been intensely used as a model to study plant mitosis and cytokinesis. Indeed, mutations in numerous genes cause defects going through PMC meiosis, PMI (pollen mitosis I) or PMII (pollen mitosis II). For example, mutation in the *TIO* (*TWO-IN-ONE*) gene, coding for a FUSED Ser/Thr protein kinase, also shows cytokinesis defects at PMI, producing binucleate pollen due to an incomplete cell plate formed during cytokinesis [9]. TIO, which is localized at the midline of the nascent phragmoplast, interacts with the PAKRP1/kinesin-12A protein, suggesting its role in cytokinesis microtubule organization [10]. In yeast two-hybrid assays, TIO also interacts with AtNACK1/TETRASPORE [11], another kinesin known to function in plant cytokinesis [12]. Indeed, *TES* and *HINKEL* [13] are two redundant kinesins involved in PMI cytokinesis [14]. They are orthologous to the tobacco *NACK1*, involved in somatic cytokinesis. NtNACK1 is upstream of a MAPK signaling cascade, where it activates the MAPKKK NtNPK1 (AtANP1, 2, 3). Once activated, NtNPK1 activates the MAPKK NtNQK (AtMKK6), and finally, NtNQK activates the MPK NtNRK1/NTF6 (AtMPK4) [15]. Activated NtNTF6 then promotes phragmoplast expansion by phosphorylating MAP65 and other Microtubule-Associated proteins (MAPs) [16].

MAPK signaling is widely used to transduce signals from cell to cell, and plants own the largest MAPK family among all eukaryotes. For example, the genome of *A. thaliana* contains > 60 MAPKKKs divided into three families: the Rafs, the Ziks, and the MEKKs, 10 MAPKKs (MKKs), and 20 MAPKs (MPKs) [17]. MAPKs have been shown to be involved in numerous processes, such as stress response [18], defense [19], hormone signaling [20], and development [21]. This last process is probably the least investigated, since relatively little is known, especially in gametophyte development. In *A. thaliana*, from the 21 MEKKs, two functionally redundant MEKKs have been found to play a role in pollen development: *map3kε1; map3kε2* (*mapkkk7; mapkkk6*) double mutants show defects in plasma membrane formation in developing pollen, and also in embryo development at the globular or heart stages [22]. Embryo development is also affected in *yoda* mutants, demonstrating the role of this MAPKKK in the asymmetric division of the zygote after fertilization [23]. Some MPKs have also been found the play roles in gametophyte development, like *AtMPK4*, involved in the cell plate expansion in male-specific meiotic cytokinesis [24, 25], or *AtMPK3* and *AtMPK6*, which function redundantly in anther and ovule integument development [26, 27], early pollen development [28], and pollen tube elongation and guidance prior to fertilization [29, 30]. Recently, two MPKs in tomato, *SlMPK7* and *SlMPK20*, were knock-down or knock-out, respectively, that affect pollen development [31, 32]. *SlMPK7* mutants displayed abnormal pollen formation, probably due to its involvement in tapetum degradation [31], while *SlMPK20* is involved in post-meiotic development through the regulation of gene expression that mediates sugar and auxin metabolisms [32].

In *S. chacoense*, a diploid species close to the cultivated potato (*S. tuberosum*), two MAPKKKs were shown to play roles in both female and male gametophyte development [33–35]. These MAPKKKs are members of a recently characterized MEKK class called the Fertilization-Related Kinases (FRKs) class, that showed rapid evolution and expansion in solanaceous species [36]. *ScFRK1* downregulated mutant [34] and *ScFRK2* overexpressed mutant [33] led to the production of small fruits with severely reduced seed set. Megagametogenesis and microgametogenesis showed development arrest at the female gametophyte stage (Mazin B., et al., unpublish). In *Arabidopsis*, *AtMAPKKK19*, *20* and *21* are their closest orthologs. In recent years, the roles of *AtMAPKKK20* have started to be uncovered. *AtMAPKKK20* was shown to interact with calmodulins and calmodulin-like proteins [37], and might be involved in pollen development, being highly expressed in pollen and as a target of the DUO1 R2R3 myb transcription factor [38]. In *Brassica napus*, *BnMAPKKK20* and *BnMAPKKK19* were shown to be stress regulated [39]. Yeast two-hybrid assays also showed that BnMAPKKK19 interacted with BnMKK8 and BnMKK9 whereas BnMAPKKK20 interacted strongly with BnMKK3 and to a lesser extent to BnMKK8 and BnMKK9 [39]. More recently, *AtMAPKKK20* was shown to modulates abscisic acid responses through the MKK5-MPK6 kinase cascade and is also involved in *Arabidopsis* cortical microtubule functions through two non-complementary pathways, one with MKKK20, MKK3 and a still unknown MPK; the second, as a non-canonical MAPK cascade made of MKKK20 and MPK18, bypassing the need for an MKK intermediate.

Herein, we provide the characterization of the MAPKKK *ScFRK3*, a third member of the FRK family in *S. chacoense* that is phylogenetically close to *AtMAPKKK19, 20 and 21.* We describe its roles in both male and female gametophyte development, where *ScFRK3* acts as a major role during gametophyte development. We also provide a putative MAPK signaling cascade involving ScFRK3, ScMKK3 and ScMPK13 in reproductive development.

## Methods

### Plant material and transformation

*Solanum chacoense* Bitt. plants (genotype G4, S_12_S_14_ self-incompatibility alleles and V22 S_11_S_13_ self-incompatibility alleles) were derived from *S. chacoense Bitt.* lines PI458314 and PI230582 from the NRSP-6 United States Potato Genebank (Sturgeon Bay, Wisconsin) and were greenhouse-grown under long-day condition (16 h light/8 h dark). For crosses, genotype V22 was used as the pollen donor. Transgenic plants were produced in the G4 genotype by agroinfiltration and callus regeneration as described previously [40] using *Agrobacterium tumefasciens* strain LBA4404. DNA cloning was done with GATEWAY® technology using pDONR™/Zeo as the entry vector and pK7GWIWG2(I) for RNAi mutant plants [41] as destination vectors. RNAi expression was under the control of the CaMV35S promoter [31, 34]. After in vitro selection on appropriate antibiotics, twenty independent plants were chosen for further analyses. Polyploid plants were identified by stomatal guard cell chloroplasts number and discarded. Semi-quantitative RT-PCRs using the Moloney Murine Leukemia Virus Reverse Transcriptase (Invitrogen) were performed with 2.5 μg of total RNA from stamens and ovules extracted from 6 mm growing buds and ovules of wild type and each mutant. All primers used for PCRs and the number of cycles for each gene are listed in Additional file 1: Table S1.

### RNA expression and in situ hybridization

RNA from different tissues (leaf, stem, root, petal, anther, ovary, style and bud) were extracted with TRIzol™ reagent (Invitrogen). RT-PCRs were performed as described earlier [42]. In situ hybridizations were performed on 6 mm flower buds. Tissue sections of 8 μm were made as described in Lantin et al. (1999) [43] with VistaVision Histobond microscope slides (VWR). Sense and antisense RNA probes were synthesized from a 600 bp *ScFRK3* PCR amplicon that included the variable C-terminal domain (for primers, see Additional file 1: Table S1) using digoxigenin-11-UTP with T3 (for sense probe) and T7 (for antisense probe) RNA polymerases (Roche). Microscope slides were hybridized with 50 ng of sense or antisense RNA probes corresponding to the best signal to ratio concentration. Probe hybridizations were made overnight at room temperature, while detection with the anti-DIG antibody was made for one hour at 37 °C. Tissue section staining was performed overnight at room temperature using NBT/BCIP solution (Roche).

### Ovule clearing and microscopy

Ovules from 3 to 8 mm buds and from mature flowers were extracted from ovaries and were fixed and cleared as described previously [44]. Cleared ovules were observed by differential interference contrast microscopy using a Zeiss Axio Imager M1 microscope with a Zeiss Axiocam HRc camera.

### Pollen development and viability analyses

Microspore development was studied by staining squashed anthers from all development stages with the Hoechst 33342 nuclear stain (NucBlue® Live ReadProbes® Reagent; Life Technologies). Developing pollen grains were observed under ultraviolet wavelength (358 nm) using a blue/cyan filter as well as under bright-field with 1% acetocarmine for pollen viability assessment. Pollen viability was also analyzed with fluorescein diacetate (FDA) staining as described previously. For pollen viability test, three replicates each using pollen from six different flowers were used. Observations were made with a Zeiss Axiocam HRc camera on a Zeiss Axio Imager M1 microscope. For SEM, fresh mature pollen grains were directly observed with a JEOL JSM-7400F High Resolution Field Emission Scanning Electron Microscope in extended vacuum mode.

### MKKs and MPKs retrieval and RACEs

Pollen- and ovule-expressed MKKs and MPKs were retrieved from a de novo ovule and pollen tube transcriptome from *S. chacoense* (Transcriptome Shotgun Assembly, Genbank accession: GDZW00000000. Amino acid sequences from the kinase domains of the *A. thaliana* 10 MKKs and 20 MPKs were used to screen against the *S. chacoense* transcriptome. The BLAST (tblastn) algorithm was used to compare the *A. thaliana* sequences against the *S. chacoense* database. The matching transcripts were then compared with the NCBI database to ensure their correspondence to true MKKs or MPKs. Since most of the transcripts lacked a complete 5′ or 3′ fragment, full-length RNA was obtained using ligase-mediated Rapid Amplification of cDNA Ends (RLM-RACEs) (GeneRacer Kit, Life Technologies).

### Directed yeast-two hybrid assays

The ScFRK3 and ScMPKs were cloned into the pGBKT7 vector which contains the binding-domain (BD) while the MKKs were cloned into pGADT7 vector which contain the activation-domain (AD) (Clontech). Individual baits (ScFRK3 or ScMKK3) and preys (respectively all MKKs or all MPKs) were respectively introduced in the Y2H Gold and Y187 yeast strains. Baits and preys were grown in SD (−)Trp and SD (−)Leu media, mixed in 2X YPAD and incubated for 16 h at 30 °C with slow shaking. One to two microliters (μL) from each mating was then deposited on selective media ([SD (−)Leu (−)Trp]; [SD (−)Leu (−)Trp (−)His (−)Ade (+)Aureobasidin A]; [SD (−)Leu (−)Trp (−) His (−) Ade (+)Aureobasidin A (+) 50 mM 3AT]) and incubated at 30 °C for 3 to 6 days. Yeast two hybrids assays were done in triplicates.

### Subcellular localization and bimolecular fluorescence complementation assays

For subcellular localization, *ScFRK3*, *ScMKK3* and *ScMPK13* ORFs were fused to the N-terminus of green fluorescent protein (GFP) using the GATEWAY® technology in the pMDC83 plasmid as the destination vector [45]. For BiFC, pUC-SPYNE and pUC-SPYCE destination vectors were used to fuse the N (pUC-SPYNE) part of the yellow fluorescent protein (YFP) or the C (pUC-SPYCE) part of the YFP to the C-terminus end of one of the proteins of interest [46]. Tagged proteins were transiently expressed in onion epidermal cells through DNA-coated microparticle bombardment using the Biolistic PDS-1000/He Particle Delivery System (Bio-Rad). Onion epidermal cells (~ 1 cm^2^) were bombarded with 1.0 μm gold particles from a distance of 9 cm and at a pressure of 1100 psi. For subcellular localization assays, as well as BiFC interactions, all experiments were done in triplicates with at least 10 fluorescent cells per replicate in order to validate the results.

## Results

### ScFRK3, a MEKK related to the *Arabidopsis* MAPKKK19, 20 and 21

**Fig. 1:**
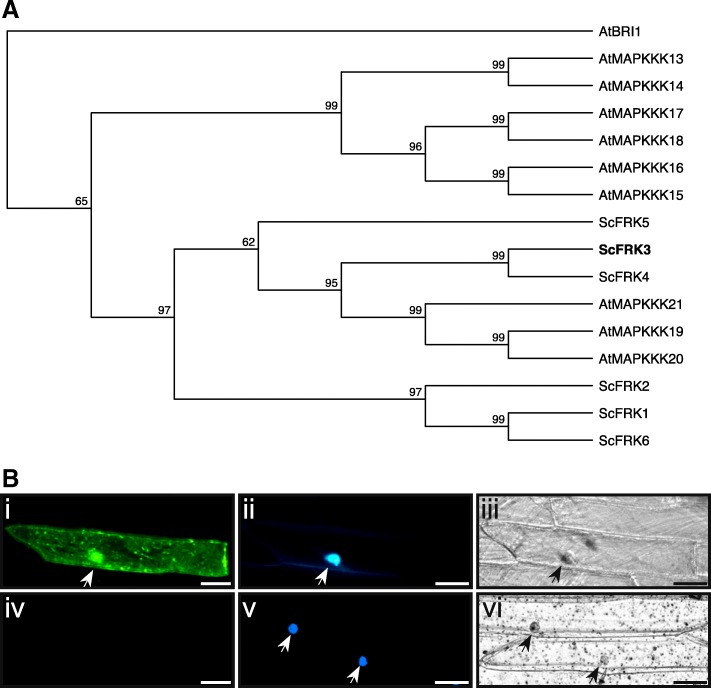
Phylogenic positioning of ScFRK3 inside the FRK class and cellular localization. **a**. Phylogenetic tree of the ScFRKs (ScFRK1–6) and AtMAPKKK13–21. ScFRK3 and ScFRK4 are the closest orthologs of the *Arabidopsis* MAPKKK19–21. The tree was made by the Neighbor Joining method using 1000 replicates and rooted with the kinase domain of AtBRI1. **b**. (i) Transient expression of *ScFRK3-GFP* in onion cells through microparticle bombardment showing the subcellular localization of ScFRK3 in the cytoplasm and nucleus. Nuclear localization confirmation of (ii) with Hoechst 33342 DNA stain. (iii) DIC image of (i and ii). (iv-vi) Negative control with (iv) bombardement of empty vector (pMDC83); (v) Nuclear localization of (iv) with Hoechst 33342 DNA stain; (vi) DIC image of v and vi. Arrowhead points to the nucleus. Scale bars: 50 μm

From the combined *S. chacoense* ovule and pollen tube transcriptome, 21 different MEKKs were isolated with six of them (ScFRK1–6) belonging to the FRK class [36]. Among these, ScFRK3 and 4 are the closest orthologs of the *A. thaliana* MAPKKK19, 20 and 21 (Fig. 1a), sharing between 43 and 75% amino acid pairwise identity with those from *A. thaliana*. The FRK class kinase has recently been shown to have largely expanded in solanaceous species [36]. In fully characterized solanaceous genomes, like *Solanum tuberosum* and *Solanum lycopersicum*, their total number (15 and 17 FRKs, respectively) account for more than 40% of the whole MEKKs subfamily. As for other members of the FRK class, ScFRK3 is a small MEKK with a 352 amino acids sequence (39 kD) consisting mainly of a protein kinase domain (from 3 to 260) and a short C-terminal domain (from 261 to 352). Unlike ScFRK1 [34], and some other members of the FRK class [36] that are predicted as nuclear proteins, ScFRK3 did not harbor a mono- or bipartite nuclear localization signal. Nonetheless, ScFRK3 was found both in the cytoplasm and the nucleus, even when linked to the GFP fluorescent marker (Fig. 1b).

### *ScFRK3* expression pattern suggests a role in reproductive development

**Fig. 2:**
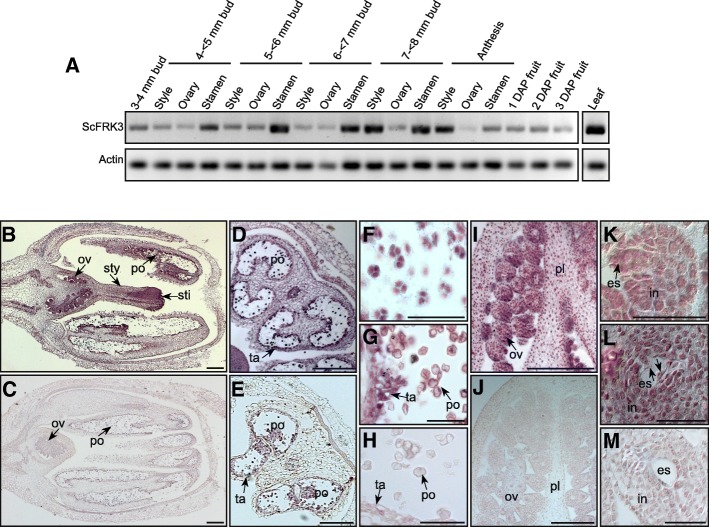
*ScFRK3* expression pattern in reproductive development. **a**. Semi-quantitative RT-PCRs on reproductive tissue using *ScFRK3* specific primers. **b**-**m**. In situ localization of *ScFRK3* transcripts using 50 ng of antisense probe in young flower bud (**b**), young stamen (**d**), pollen at tetrad stage (**f**) and young microspore stage (**g**), inside the ovary (**i**), in ovule before FM stage (**k**) and in ovule after the first mitosis (**l**). In situ hybridizations using 50 ng of sense probe in young flower bud (**c**), young stamen (**e**), young microspore (**h**), inside the ovary (**j**) and an ovule after the first mitosis (**m**). Scale bars: 200 μm (**b**-**e**, **i** and **j**) and 50 μm (**f**-**h**, **k**-**m**). Abbreviations: es, embryo sac; in, integument; ov, ovule; pl, placenta; po, pollen; sti, stigma; sty, style; ta, tapetum; nu, nucleus

It was recently shown that *ScFRK3* was expressed in somatic tissues such as leaves, stems, roots, as well as in flower buds [36], suggesting a role in reproductive development similarly to the two previously described FRK class members, *ScFRK1* and *ScFRK2* [33–35]. To precisely determine in which tissue and developmental stages *ScFRK3* is expressed, semi-quantitative RT-PCRs were performed on flower buds ranging from 3 to 8 mm length, on flowers at anthesis, and on fruits from one to three days after pollination (DAP). As shown in Fig. 2a, *ScFRK3* is expressed in reproductive tissues of very young buds (3 mm length). While *ScFRK3* expression in ovary remains unchanged at all stages, stylar expression increased in 7–8 mm buds (corresponding to 1 day before anthesis; 1 DBA). Stamen mRNA expression was the strongest overall with a steep increase in 5–6 mm buds that stayed stable until the day before anthesis. At anthesis stage, stamen expression decreased sharply while stylar expression remained high. *ScFRK3* mRNA was also detected in young fruits, from 1 to 3 DAP.

To finely define the *ScFRK3* expression pattern, in situ hybridizations were performed on developing flower buds. As shown in Fig. 2b, *ScFRK3* is expressed in 6 mm whole flower buds, especially in the ovules, pollen and tapetum. *ScFRK3* mRNAs are detected in young pollen, particularly in tetrads (Fig. 2d and f) and young microspores before PMI (Fig. 2g). Inside the ovary, *ScFRK3* is highly expressed in the ovule compared to the placenta and pericarp (Fig. 2i), although expression is still lower than in pollen. *ScFRK3* ovular expression is observed in the integument as well as in the young embryo sac. As shown in Fig. 2k and l, *ScFRK3* mRNAs are detected in the ovule at the MMC stage (Fig. 2 k) as well as during the first mitosis of the FG (Fig. 2l). Taken together, *ScFRK3* expression profile strongly suggests a role in gametophyte development, especially at young developmental stages.

### *ScFRK3* RNAi plants show reproductive defects

**Fig. 3:**
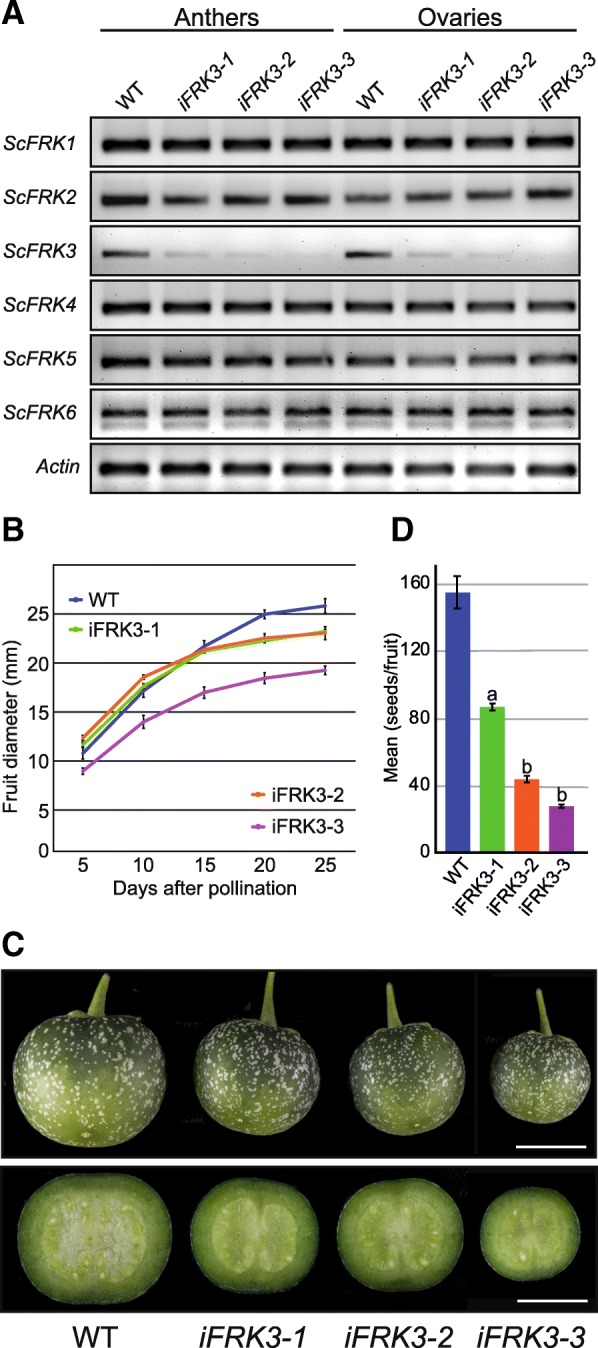
*iFRK3* mutants produce smaller fruits and lesser seeds. **a**. RT-PCR analysis of *ScFRK1–6* transcripts on anthers and ovules taken from 6 mm flower buds of WT and *iFRK3* mutant plants. **b**. Fruits diameters from WT and *iFRK3* mutant lines from 5 DAP to 25 DAP (maturity). Ten fruits per plant lines were used for student test. At 25 days, each *ScFRK3* RNAi mutants showed a significaly different size (*p* < 0.001; student test) compare to the WT. **c**. Comparison of fruit morphology and seed number of control plants and *iFRK3* mutant. D. Number of seeds per fruit in *ScFRK3* RNAi mutants. Five fruits per plant lines were used for a: student test *p* < 0.02; b: student test *p* < 0.001

To elucidate the function of the *ScFRK3* gene, RNAi mutant plants were generated and tested for the downregulation of *ScFRK3* mRNA expression levels. RT-PCRs were performed on stamens and ovules extracted from 6 mm growing buds (Fig. 3a). Three different lines (*iFRK3–1*, *iFRK3–2* and *iFRK3–3*) that showed a significant decrease in *ScFRK3* mRNA levels in anthers and ovule during development were kept for further investigation. Since pairwise nucleotide sequence identity between *ScFRK3* and its five *S. chacoense* closest relatives (Fig. 1) ranged from 40 to 69%, semi-quantitative RT-PCR analyses were performed to determine whether expression of the other five *ScFRK* members may be affected by the *ScFRK3* RNAi construct. As shown in Fig. 3a, RNA expression levels from the five other *ScFRKs* remained unchanged in all three *ScFRK3* RNAi mutants tested, indicating that the reproductive phenotype observed in *ScFRK3* RNAi lines is linked to the downregulation of the *ScFRK3* gene only. The three *ScFRK3* RNAi lines were then cross-pollinated with a compatible *S. chacoense* accession for fruit size and seed set analyses. Lines i*FRK*3–1, i*FRK3*–2 and i*FRK3*–3 showed severely reduced fruit size (Fig. 3 b and c). Seed set in these three interference lines was also severely reduced, with a mean ranging from 28 to 87 seeds compared to 155 in WT plants (Fig. 3 c and d).

### Ovule development is affected in *ScFRK3* RNAi mutants

**Fig. 4:**
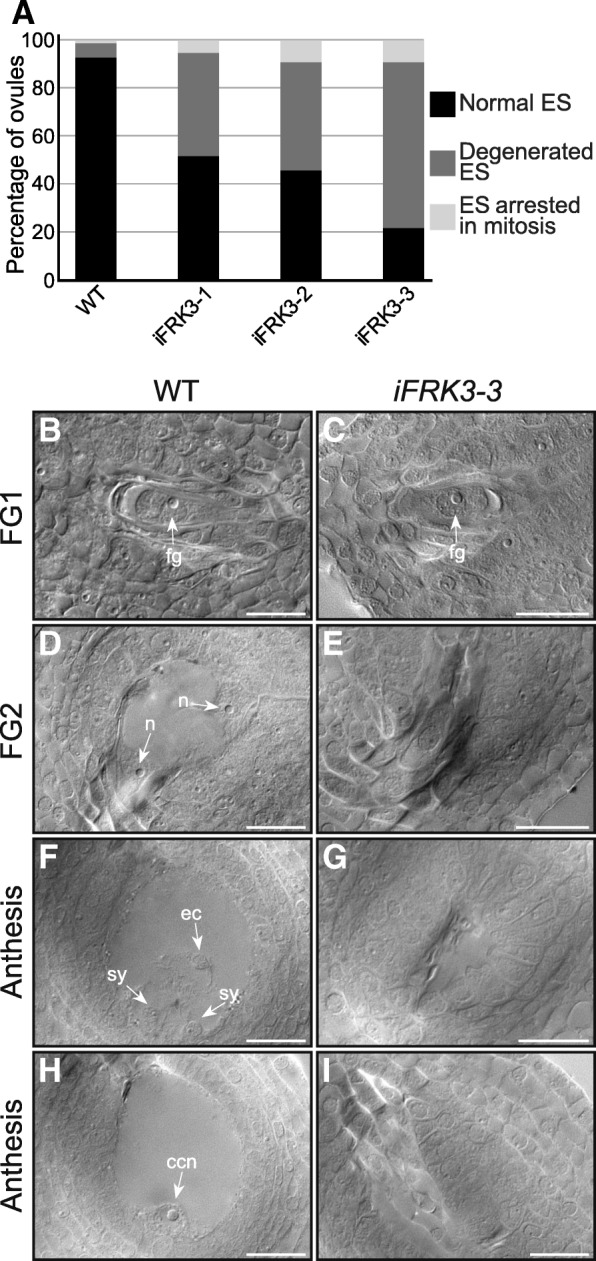
Ovule phenotyping in *iFRK3* mutants. **a**. Percentage of normal embryo sacs, degenerated ES (dark gray) and ES arrested during mitosis stages (light gray) from anthesis flowers in WT and *iFRK3* mutants. **b**-**i**. DIC images of developing ovules in WT and *iFRK3–3* mutant plants. Ovule at the FG1 stage in WT (**b**) and *iFRK3–3* mutant flowers (**c**). Ovule at first mitosis stage (FG2) in WT (**d**) and degenerating in *iFRK3–3* mutant plants (**e**). Mature ovule in WT (F and H) and in *iFRK3–3* mutant flowers (**g** and **i**). F and H are Z-stack of the same ovule. Abbreviations: ccn, central cell nucleus; ec, egg cell; fg, functional megaspore; n, nuclei; sy, synergids. Scale bars: 20 μm

Since *ScFRK3* expression is detected early on during reproductive development (Fig. 2) and seed set clearly affected in RNAi *ScFRK3* mutant lines (Fig. 3 c and d), we microscopically observed gametophytic development on cleared ovules. As for other solanaceous species, *S. chacoense* has a Polygonum-type embryo sac (ES). To determine at which stages the ovule is mostly affected in RNAi mutant lines, ovules from WT and mutant flower buds (from 3 to 8 mm length) were analyzed. The results are summarized in Table 1. Microscopic analyses of ~ 3–4 mm WT bud showed high proportion of ovules (red) at the MMC stage, with a low proportion (yellow-white) of ovules beginning the meiosis. In contrary, in ~ 4–5 mm buds the majority of ovule (red) pass the meiosis stage (functional megaspore) and began mitosis. After the three mitosis, an eight nuclei ES (FG5-FG6) is observed in ~ 6–7 mm buds. Central cell karyogamy, cellularization and antipodal cells degeneration are the final steps of ES maturation (FG7), occurring generally in ~ 7–8 mm buds, approximately 1 day prior anthesis. These three steps are grouped together and represented by Mat (for maturation) in Table 1. Ovules from the three *iFRK3* mutants started to develop normally but, unlike in WT, most of ES did not progress further than the functional megaspore stage (FM) (represented in red compartments, Table 1). A small proportion of the ES, however, displayed delayed development, and could progress through one, two or the three mitosis. Figure 4a shows the proportion of normal ES (black), degenerated ES (dark grey) and ES that were stopped during one of the mitosis stages (light grey) in *iFRK3* mutants and WT flowers at anthesis. In mature flowers, 93% of WT ES were normal while only 52, 46 and 22% of i*FRK*3–1, i*FRK3*–2 and i*FRK3*–3 ES were normal, respectively. In contrast, only 6% of WT ES were completely degenerated, compared to 43, 45 and 69% in i*FRK*3–1, i*FRK3*–2 and i*FRK3*–3, respectively. Typical phenotypes presented in *iFRK3* mutants are shown in Fig. 4 (b-i). Figure 4 shows that the FM development is not affected in *iFRK3* mutants (Fig. 4b and c), unlike the first mitosis step or FG2 (Fig. 4d and e), leading to the degeneration of the female gametophyte in mature flowers (Fig. 4f-i). These results suggest that *ScFRK3* is involved in female gametophyte development.

**Table 1:**
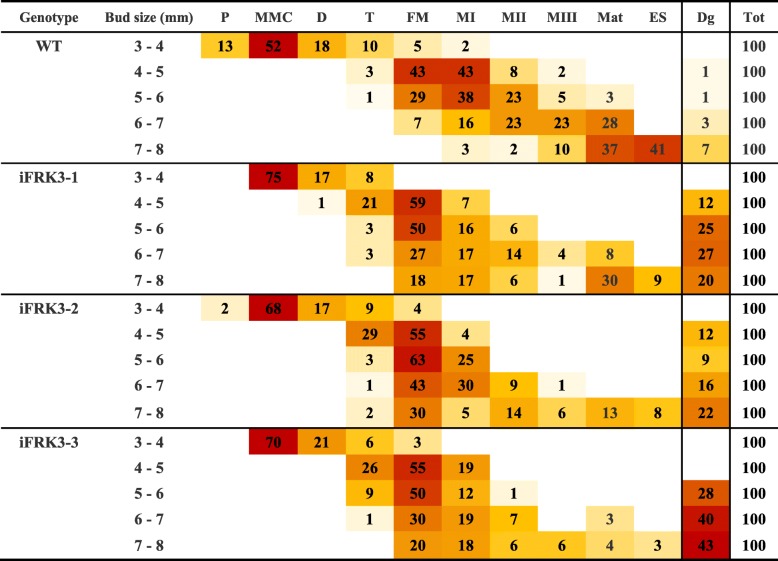
Correlation between ovule development stages and bud size in WT and *iFRK3* mutants

*P* Primordium, *MMC* Megaspore Mother Cell, *D* Dyad, *T* Tetrad, *FM* Functional Megaspore, *MI* Mitosis I, *MII* Mitosis II, *MIII* Mitosis III, *Mat* Embryo sac Maturation, *ES* Embryo Sac, *Dg* Degenerated, *Tot* Total of ovules observed. Expression levels are shown on a color scale with red indicating strong proportion of ovule, yellow for low proportion of ovule, and white with no ovule at a given stage

### Pollen development and viability are also affected in *ScFRK3* RNAi lines

**Fig. 5:**
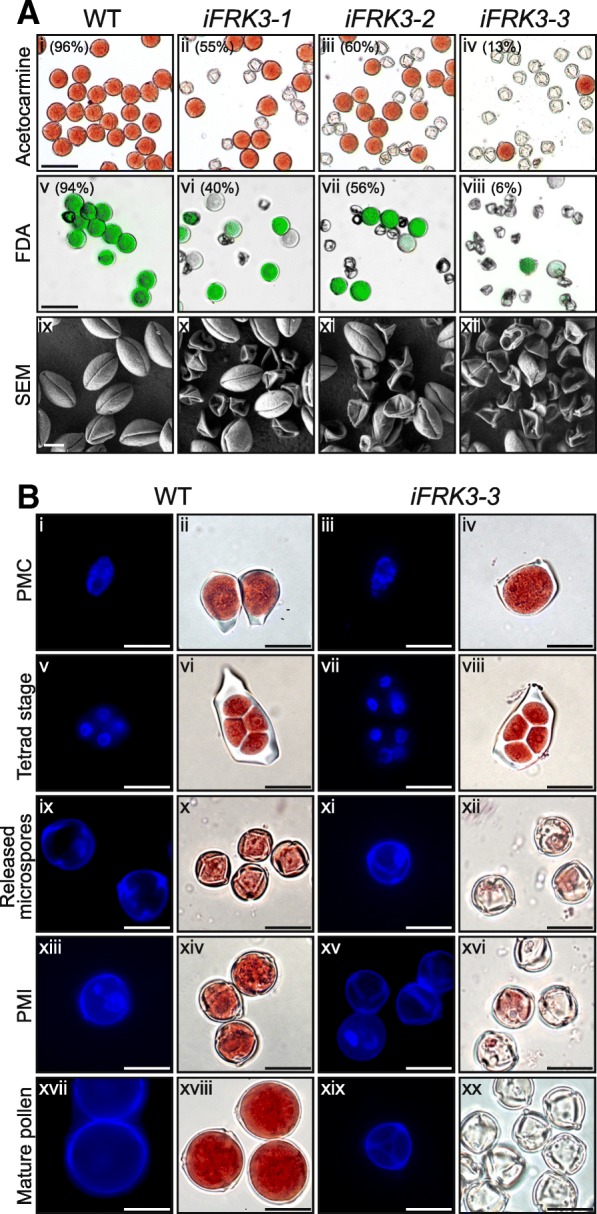
Pollen phenotyping in *iFRK3* mutants. **a**. The pollen grains were examined by acetocarmine staining (i-iv), fluorescein diacetate (FDA) staining (v-viii) and scanning microscopy, respectively (ix-xii). For acetocarmine and FDA scale bars: 50 μm. For SEM scale bars: 10 μm. **b**. Pollen development phenotyping using Hoechst 33342 DNA stain (i, iii, v, vii, ix, xi, xiii, xv, xvii and xix) and acetocarmine staning (ii, iv, vi, viii, x, xii, xiv, xvi, xviii and xx) in WT (i, ii, v, vi, ix, x, xiii, xiv, xvii and xviii) and *iFRK3–3* mutant (iii, iv, vii, viii, xi, xii, xv, xvi, xix and xx) plants. PMC stage (i-iv). Tetrad stage (v-viii). Released microspores (ix-xii). Microspores after PMI (xiii-xvi). Mature pollen (xvii-xx). Scale bars: 20 μm

Since *ScFRK3* mRNAs were detected in stamen, pollen viability was also analyzed in *iFRK3* mutants. Pollen from WT, i*FRK*3–1, i*FRK3*–2 and i*FRK3*–3 mature flowers were stained with 1% acetocarmine solution, and viable as well as non-viable pollen grains were counted. As displayed in Fig. 5a, 96% of WT pollen grains were viable, compared to 55, 60, and 13% in i*FRK*3–1, i*FRK3*–2 and i*FRK3*–3 respectively. With the acetocarmine staining method, viable pollen grains are round and evenly stained in pink (Fig. 5a, i-iv), while dead pollen grains are smaller, translucent and mostly deformed. For more accuracy, fluorescein diacetate (FDA) staining was also used to analyze pollen viability in WT and *iFRK3* mutants (Fig. 5a, v-viii). In comparison to WT, around half of the pollen grains were considered dead, failing to show FDA fluorescence in i*FRK3*–1 and i*FRK3*–2 mutants. For i*FRK3*–3, only 6% of the pollen grains showed FDA fluorescence and were considered alive. To analyze the ultrastructure of pollen defects in *iFRK3* plant lines in more details, pollen grains were observed by scanning electron microscopy. Normally, *Solanum chacoense* pollen grains consists of three equally distributed apertures that run longitudinally to the pollen axis. However, *iFRK3* mutants produced numerous shriveled and collapsed pollen grains (Fig. 5a, ix-xii). As for ovule development, pollen viability phenotype was also more severe in the *iFRK3–3* line.

To determine when exactly pollen development was affected in *ScFRK3* disrupted plants, pollen viability assays and DNA staining were performed on developing WT and mutant pollen. A comparison between WT and *iFRK3–3* plants is shown in Fig. 5b. At the PMC stage, pollen mother cells from WT and *iFRK3–3* plants were fully viable, as observed by acetocarmine staining (Fig. 5b, i-iv). Next, at the microspore tetrad stage, pollen from both WT and *iFRK3–3* plants had fully undergone meiosis (Fig. 5b, v-viii), including microspore release (Fig. 5b, ix-xii). As the microspores grew, PMI occurred normally in the WT and produced a two-celled pollen grain, the vegetative and the germ cells (Fig. 5b, xiii and xiv). In contrast to WT pollen, PMI did not occur in most pollen grains in the *iFRK3–3* mutant, already starting to collapse and die (Fig. 5b, xv-xvi). WT mature pollen grains (Fig. 5b, xvii and xviii) were fully viable as observed with by acetocarmine staining, while pollen grains from *iFRK3–3* mutants were mostly dead (Fig. 5a, iv and b, xix and xx). The few remaining pollen grains that could go through PMI grew successfully to maturity, as for WT pollen. These results show that *ScFRK3* is involved not only in the female gametophyte, but also on the male side, in pollen development.

### ScFRK3 interacts with ScMKK3

In the MAPKKK family, ScFRK3 is classified as a MEKK subfamily kinase, thus its involvement in a canonical MAPK signaling pathway is expected. To reconstitute a three-tier kinase module with ScFRK3 acting as the upstream kinase, yeast-two hybrid (Y2H) assays were performed with the ScMKKs and ScMPKs found in the *S. chacoense* combined ovule and pollen transcriptome. Using the 10 MKKs and the 20 MPKs from *A. thaliana* to screen the *S. chacoense* transcriptome, five ScMKKs and 16 ScMPKs were retrieved (See Additional file 1: Table S2). A phylogenetic analysis for both *S. chacoense* MKKs (Additional file 2: Figure S1) and MPKs (Additional file 3: Figure S2) was performed to assign their closest ortholog (s) in *A. thaliana* and *Solanum lycopersicum*. All ScMPKs expressed in the combined pollen and ovule transcriptome had clear orthologs in tomato, while three others (isotigs 27,038, 27,937 and 36,598) could not be clearly assigned among the 20 AtMPKs (Additional file 1: Table S2 & Additional file 3: Figure S2).

**Fig. 6:**
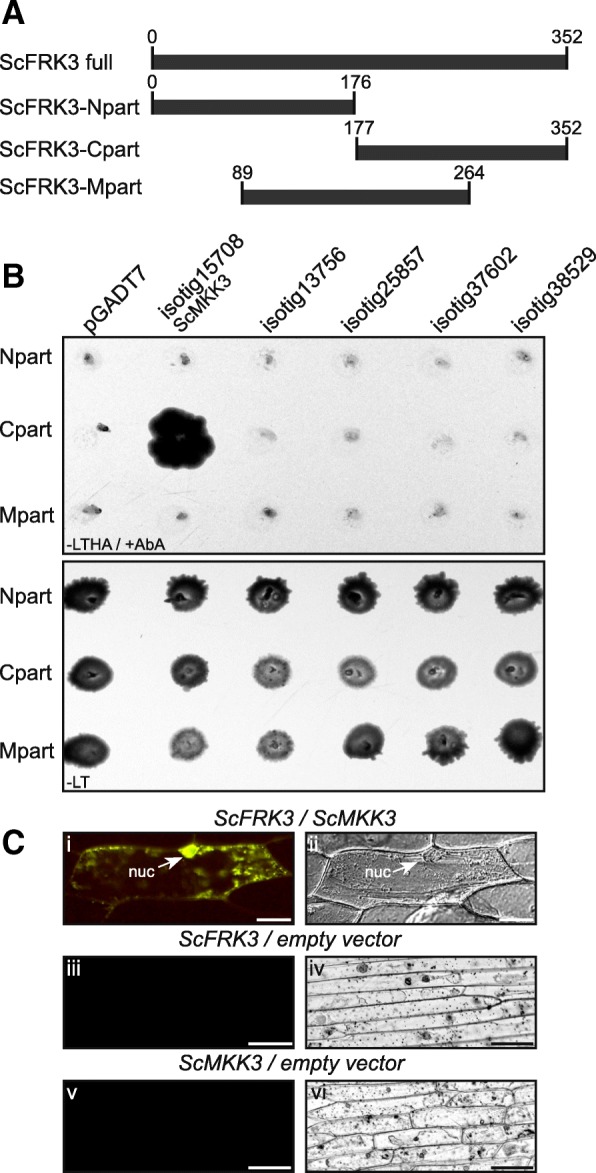
ScFRK3 interacts with the MAP kinase kinase ScMKK3. **a**. Diagram showing the three different constructs made in pGBKT7 with ScFRK3 as the insert. **b**. Directed Y2H assays between the three ScFRK3-pBKT7 constructs and the five ScMKKs found in the *S. chacoense* transcriptome. The only positive interaction is shown between ScFRK3-Cpart and ScMKK3. -LT: Y2H media without leucine and tryptophan; −LTHA/+AbA: Y2H media without leucine, tryptophan, histidine, adenine and with aureobasidin A. **c**. (i) The interaction between ScFRK3 (35S::ScFRK3-YFPN) and ScMKK3 (35S::ScMKK3-YFPC) is confirmed by BiFC in onion cells. (iii) 35S::ScFRK3-YFPN and 35S::YFPC only (empty vector) used as negative control. (v) 35S::ScMKK3-YFPN and 35S::YFPC only (empty vector) used as negative control. (ii, iv, vi) All BiFC interactions were done in triplicates with at least 10 fluorescent cells per replicate. DIC image of i, iii and v. Scale bars: 50 μm

Directed Y2H assays were then performed using ScFRK3 fused to the binding domain (BD) as bait and all ScMKKs from our combined pollen and ovule transcriptome were fused to the activation domain (AD) as preys. Since the full version of ScFRK3 was interacting with all preys, including the negative control (pGADT7, empty vector, not shown), three different versions of the ScFRK3-pGBKT7 constructs were made (Fig. 6a). The N-terminal construct (ScFRK3-Npart), comprised amino acids 1 to 176, the middle part (ScFRK3-Mpart) comprised amino acids 89 to 264, while the ScFRK3-Cpart comprised amino acids 177 to 352. While none of the separate ScFRK3 constructs interacted with the empty vector (pGADT7), the ScFRK3-Cpart interacted only with isotig15708, which was renamed ScMKK3, being the closest ortholog to the *Arabidopsis* MKK3 (Fig. 6b). This interaction was exclusive since only the ScFRK3-Cpart interacted with ScMKK3, while the ScFRK3-Npart and -Mpart did not interact with any ScMKKs. The ScFRK3-ScMKK3 interaction was also confirmed in vivo in onion epidermal cells using BiFC assays. When ScFRK3-NYFP and ScMKK3-CYFP were co-expressed in onion cells, fluorescence was observed inside the nucleus and in the cytoplasm (Fig. 6c, i and iii). Moreover, the expression pattern of *ScMKK3* was verified by semi-quantitative RT-PCR using specific primers and was found to be expressed in somatic as well as reproductive tissues (style, ovary, stamen and buds) (Additional file 4: Figure S3A), overlapping the *ScFRK3* expression pattern. As for ScFRK3, ScMKK3-GFP alone was also localized inside the nucleus and the cytoplasm (Additional file 4: Figure S3B, i and ii). Taken together, these results suggest that ScFRK3 interacts with ScMKK3 in both the nucleus and cytoplasm.

### ScMKK3 interacts with ScMPK13

**Fig. 7:**
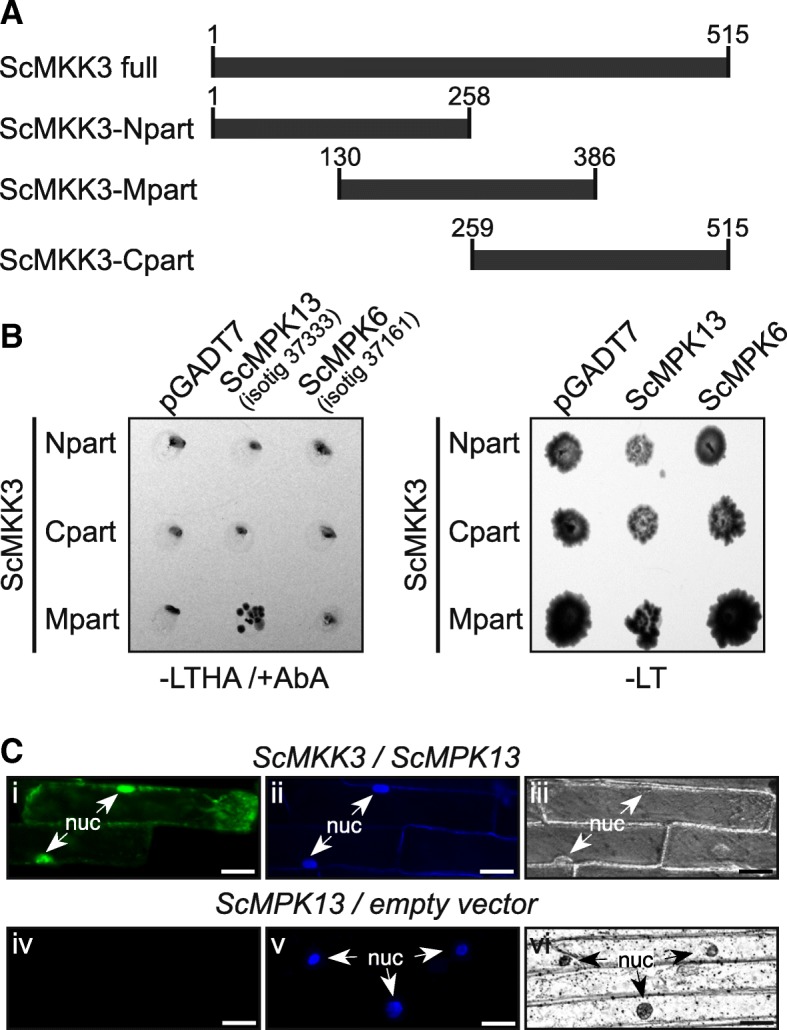
ScMKK3 interacts with the MAP kinase ScMPK13. **a**. Diagram showing the three different constructs made in pGBKT7 with ScMKK3 as the insert. **b**. Directed Y2H between the three constructions of ScMKK3-pBKT7 and two ScMPKs. The only positive interaction is shown between ScMKK3-midpart and ScMPK13. **c**. (i) The interaction between ScMKK3 (35S::MKK3-YFPC) and ScMPK13 (35S::MPK13-YFPC) is confirmed by BiFC in onion cells. (iv) 35S::MPK13-YFPC and 35S::YFPN only (empty vector) used as negative control. (ii, v) All BiFC interactions were done in triplicates with at least 10 fluorescent cells per replicate. Nuclear localization confirmation with the Hoechst 33342 DNA stain. (iii, vi) DIC image of i and ii, and iv and v, respectively. Scale bars: 50 μm

To identify MPK(s) that could act downstream of ScMKK3 and form a possible signaling cascade downstream of ScFRK3, directed Y2H assays were performed using ScMKK3 fused to the AD as bait, and all 16 ScMPKs from our combined pollen and ovule transcriptome fused to the BD as preys [36]. Since the full version of ScMKK3 was interacting with all ScMPKs preys as well as the negative control vector (pGADT7, empty vector, not shown), three new versions of *ScMKK3* were produced. The ScMKK3 constructs are shown in Fig. 7a. Amongst the 16 ScMPKs, none interacted with the ScMKK3-N and -C parts while only one interacted with the ScMKK3-Mpart, the *S. chacoense* isotig37333 (Fig. 7b). None of the other 15 ScMPKs, nor the negative control, grew on -LTHA/+AbA selective media (not shown except for isotig37161, since the 14 others were also negative). When compared to the *A. thaliana* MPKs, the closest ortholog of isotig37333 was clearly the *A. thaliana* MPK13 (Additional file 3: Figure S2), thus, isotig37333 was renamed ScMPK13 to follow the *A. thaliana* nomenclature. The Y2H interaction between ScMKK3 and ScMPK13 was also confirmed by in vivo complementation assays using BiFC in onion epidermal cells (Fig. 7c). Yellow fluorescence was observed in the nucleus and cytoplasm of cells co-expressing ScMKK3-CYFP and ScMPK13-NYFP. Like most MPKs [47], ScMPK13-GFP is localized in the nucleus and in the cytoplasm (Additional file 4: Figure S3 iii). Thus, the above-mentioned results strongly suggest that ScMKK3 and ScMPK13 interact together in a potential signaling cascade downstream of ScFRK3. As expected, RT-PCR analysis showed that *ScMPK13* is also expressed in buds, and in other reproductive tissues (Additional file 4: Figure S3A), overlapping the *ScFRK3* and *ScMKK3* expression patterns, supporting the hypothesis that ScFRK3-ScMKK3-ScMPK13 form a likely three-tiered MAPK cascade.

## Discussion

Plant genomes harbor the largest MAPK family within the eukaryotes, suggesting major roles and a wide involvement in various biological processes. Here, we introduced *ScFRK3*, a *Solanum chacoense* MAPKKK within the MEKK family. *ScFRK3* is part of the FRK class (Fertilization-related kinases) of which two other members have been previously characterized [33–35]. The FRK class is a monophyletic group inside the MEKK subfamily that appeared within the *Angiospermae* and has largely expanded within the *Solanaceae* family [36]. The FRK class can be further subdivided into 4 groups. Compared to *A. thaliana* (*Brassicaceae*) and *G. raimondii* (*Malvaceae*), that respectively have three and two FRK class kinases, *S. tuberosum* and *S. lycopersicum* (*Solanaceae*) possess 15 and 17 members, respectively. Furthermore, of the four FRK groups found in the *Solanaceae*, *A. thaliana* and *G. raimondii* only harbor one, group 4, the most ancient group in dicots [36]. The highly specific tissue expression for the first two FRKs characterized and the phenotypes observed in *ScFRK2* overexpression plant lines [33, 35] and RNAi down-regulated *ScFRK1* lines [34], showed that these kinases play important roles in reproductive tissues, suggesting that other members of the FRK class might also play roles in reproductive development. The current analysis of the *ScFRK3* gene supports this hypothesis. As for *ScFRK1* and *2*, *ScFRK3* is expressed in developing ovules and stamens, which is consistent with the developmental defects observed in both male and female gametophytes in *ScFRK3* down-regulated RNAi mutants. But, contrary to *ScFRK1* and *ScFRK2*, *ScFRK3* is also expressed in leaves to similar levels than in reproductive tissues, while no obvious leaf phenotype could be observed in RNAi lines that showed severe reproductive defects. Since no single mutants could be rescued by the other FRKs, *ScFRK1*, *2* and *3* are clearly not genetically redundant. Considering that the amino acid pairwise identity between *ScFRK1 to 3* ranges from 32 to 42% (42 to 57% nucleotide identity from the ORF), it is not surprising that no RNA interference between *ScFRK1*, *2* and *3* was observed, as shown in Fig. 3. Furthermore, although *ScFRK1* to *3* are expressed in both male and female reproductive tissues, their respective mRNA expression does not follow the same pattern. *ScFRK1* mRNA expression is detected very early on during ovule and pollen development, in the integument and megaspore mother cell (MMC) and in pollen mother cells (PMC), respectively. Before anthesis, *ScFRK1* mRNA levels increase in ovaries to reach a maximum around anthesis, were strong accumulation of *ScFRK1* mRNAs can be observed in the integument, the synergids and egg cell. In the absence of pollination, *ScFRK1* mRNA levels slowly decrease, being still high 72 h after anthesis. Pollination and fertilization have drastic effects on *ScFRK1* mRNA accumulation with a steep decrease already 12 h after pollination, and an almost complete absence of *ScFRK1* mRNAs at fertilization [34]. On the contrary, *ScFRK2* mRNA levels are very weak in all tissues tested and almost undetectable in mature ovaries, where they increase dramatically after fertilization [33]. *ScFRK3* mRNAs, however, are more abundant in developing stamens than in developing ovules. *ScFRK3* RNA level is slightly higher in ovules of 5–6 mm buds and decreases to reach undetectable expression (in RT-PCR) at anthesis, while expression is still detected in stamens. Weak expression is still detected after pollination and in developing fruits. In situ hybridization confirmed the RT-PCR results, showing that *ScFRK3* is expressed strongly in developing pollen (tetrads and microspore) as well as in the anther tapetum, and is also expressed in young ovules, at lower levels.

Unlike the other two FRKs characterized previously, ScFRK3 is found both in the cytoplasm and the nucleus, where it possibly initiates its signaling cascade. Yeast two hybrid screens revealed that only ScFRK3 (Fig. 6), and not ScFRK1 nor ScFRK2 (data not shown), could interact with ScMKK3. Although the full ScFRK3 sequence interacted with all prays and negative control, segmentation in three parts showed that only the ScFRK3 C-terminal part interacted with ScMKK3 (Fig. 6). Using Y2H and BiFC assays, the interaction between AtMKKK20 and AtMKK3 showed that the AtMKKK20 C-terminal part comprised a motif enabling a strong interaction with the AtMKK3, supporting the interaction between ScFRK3 and ScMKK3 in *S. chacoense* [48]. Furthermore, fine dissection of the AtMKKK20 C-terminal part pinpointed to a smaller segment that is also present in AtMKKK19 and 21 as well as ScFRK3 and 4 (Additional file 5: Figure S4). This segment encompassed a typical DEF mammalian MAP kinase docking site (Docking site for ERK, FXFP), strongly supporting its interaction with ScMKK3. Such DEF domains are generally characterized by an FXF [P/D/E)] motif located between 6 and 20 amino acids C-terminal to the [S/T]-P phosphoacceptor site [49]. Of the 16 ScMPKs retrieved from the *S. chacoense* pollen and ovule transcriptome, only one, isotig37333 renamed ScMPK13, being closest to the *A. thaliana* AtMPK13, interacted with ScMKK3 (Fig. 7). Both *ScMKK3* and *ScMPK13* genes are also co-expressed in the same tissues as *ScFRK3* (compare Fig. 2a and Additional file 4: Figure S3A). Furthermore, BiFC assays showed that ScFRK3 co-localized with ScMKK3 (Fig. 6c), and ScMKK3 with ScMPK13 (Fig. 7c), both in the cytoplasm and the nucleus, suggesting the possibility of a unique signaling cascade specific to ScFRK3, involving ScMKK3 and ScMPK13.

In *A. thaliana*, several mutants have been identified for their defects in gametophyte development, especially in both male and female mitosis steps. For example, some proteins associated with microtubules and cytokinesis, like TIO in the male gametophyte, are crucial for male and female mitosis [9, 12]. One exception is the potential signaling cascade involved in PMI, which starts with HINKEL and TETRASPORE acting as receptor/sensor, transmitting signals to the MAPK cascade (ANP1–3 - MKK6 - MPK4) to phosphorylate MAP65 and other MAPs involved in microtubule organization [15]. On the female side, despite all the mutants blocked in FG1 stage, no potential signaling cascade has yet been uncovered. For example, AtKin-1, a kinesin involved in microtubule dynamics and FG mitosis, also play roles in long-distance transport of organelles, vesicles and chromosomes. Yet no interacting partner has been found [8]. In ES maturation, the CKI1 histidine kinase has been shown to act upstream of the MYB119 transcription factor, which expression level is lower in *cki* mutants [50]. Similarly, no intermediate proteins have yet been uncovered in this pathway.

Recently, a tomato MAPK from group D, SlMPK20, was shown to regulate pollen development after meiosis, with no effect on the maternal side [32]. Another tomato MAPK, SlMPK7, orthologous to AtMPK4, both classified in MAPK group B, like ScMPK13, have been reported as actors in tapetum and early pollen development [31]. Here, we showed the involvement of the *ScFRK3* in gametophyte development, a MAPKKK clearly acting in both male and female gametophyte development, that interacted only with ScMKK3. Our protein-protein interaction results suggest that the ScFRK3-ScMKK3-ScMPK13 could be a bona fide three-tiered cascade involved in plant gametophyte development. Further genetic analysis of the two downstream kinases from *ScFRK3* should reveal the full role of this new cascade.

## Conclusions

The present study on the MAPKKK ScFKR3 confirmed the implication of the ScFRK family in reproductive development. The *ScFRK3* showed strong expression during anther and ovary development, specifically in the male and female gametophyte, hence *ScFRK3* RNAi mutant plants showed severe defects in reproduction, with embryo sac and pollen development arrested before mitosis I. Protein-protein interaction assays also suggested a three-tiered MAPK cascade with ScFRK3-ScMKK3-ScMPK13. This study reinforces the important role of MAPK kinase cascades in the development of reproductive tissues in plants.

## Additional files

Additional file 1: **Table S1.** List of primers. **Table S2.** MKKs and MPKs found in *S. chacoense* ovule and pollen transcriptome and their orthologs in *A. thaliana*. MKKs and MPKs found in *Solanum chacoense* ovule and pollen transcriptomes and their closest orthologs in *Arabidopsis thaliana* and *Solanum lycopersicum* (tomato) from neighbor joining phylogenies based on their protein kinase domain from Prosite (https://prosite.expasy.org). (DOCX 32 kb)

Additional file 2: **Figure S1.** Phylogenetic tree showing the relations between the AtMKKs, the SlMKKs and the ScMKKs found in the *S. chacoense* ovule and pollen transcriptome. In tomato, the closest ortholog of the *S.chacoense* isotig15708 (ScMKK3) is Solyc03g019850, named SlMKK5 (ITAG release 3.2; http://solgenomics.net) with 99% amino acid sequence identity. The MKKs are classified in four groups, from A to D. The tree was made by the Neighbor Joining method using 1000 replicates and rooted with the *Arabidopsis* BRI1 receptor kinase using MacVector 17 software. Only the kinase domain was used for the alignment. (PDF 137 kb)

Additional file 3: **Figure S2.** Phylogenetic tree showing the relations between the AtMPKs, SlMPKs and the ScMPKs found in the *S. chacoense* transcriptome. The MPKs are classified in four groups, from A to D. The tree was made by the Neighbor Joining method using 1000 replicates and rooted with the *Arabidopsis* BRI1 receptor kinase using MacVector 17 software. Only the kinase domain was used for the alignment. (PDF 155 kb)

Additional file 4: **Figure S3.** *ScMKK3* and *ScMPK13* expression and localization. A. Expression profiling on different tissues for *ScMKK3* and *ScMPK13* using semi-quantitative RT-PCRs. B. Protein localization of ScMMK3 and ScMPK13 using microparticle bombardment. ScMKK3-GFP localization in onion cell (i) and DIC image. ScMPK13-GFP localization in onion cell (iii) and DIC image (iv). Scale bars: 50 μm. (PDF 236 kb)

Additional file 5: **Figure S4.** Conservation of the C-terminal ScFRK3 segment and its closest genes in *S. chacoense* and *A. thaliana*. The AtMKKK20 C-terminal segment (285–342) was shown to interact with AtMKK3 and harbored a typical DEF mammalian MAP kinase docking site (Docking site for ERK, FXFP) identical to the *S. chacoense* ScFRK3 and 4, as well as the two closest MKKKs in *A. thaliana* [48]. DEF domains are generally characterized by a FXF [P/D/E)] motif located between 6 and 20 amino acids C-terminal to the [S/T]-P phosphoacceptor site [49]. (PDF 269 kb)

## Abbreviations

BCIP: 5-bromo-4-chloro-3-indolyl-phosphate
BiFC: Bimolecular fluorescence complementation
DIC: Differential interference contrast
DIG: Digoxigenin
FDA: Fluorescein diacetate
FG: Female gametophyte
FM: Functional megaspore
GFP: Green fluorescent protein
kD: Kilodalton
MAP: Microtubule-associated proteins
MAPK, MPK: Mitogen-activated protein kinase
MAPKK, MKK, MEK: Mitogen-activated protein kinase kinase
MAPKKK, MKKK, MEKK: Mitogen-Activated Protein Kinase Kinase Kinase
MMC: Megaspore mother cell
MW: Molecular weight
NBT: Nitro blue tetrazolium
NLS: Nuclear localization signal
PMC: Pollen mother cell
PMI: Pollen mitosis I
PMII: Pollen mitosis II
RLM-RACE: RNA Ligase Mediated Rapid Amplification of cDNA Ends
RNA: Ribonucleic acid
RNAi: RNA interference
RT-PCR: Reverse transcription polymerase chain reaction
ScFRK3: *Solanum chacoense* Fertilization-Related Kinase 3
SEM: Scanning electron microscopy
Y2H: Yeast-two hybrid
YFP: Yellow fluorescent protein
YPAD: Yeast extract, peptone, adenine
