# Supplementary material for: The Solanum chacoense Fertilization-Related Kinase 3 (ScFRK3) is involved in male and female gametophyte development
Source: BMC Plant Biol. 2019 May 16;19:202. doi: 10.1186/s12870-019-1804-0 (PMC6524262; doi:10.1186/s12870-019-1804-0)
Supplement: Supplementary file 1 — Table S1. List of primers. Table S2. MKKs and MPKs found in S. chacoense ovule and pollen transcriptome and their orthologs in A. thaliana. MKKs and MPKs found in Solanum chacoense ovule and pollen transcriptomes and their closest orthologs in Arabidopsis thaliana and Solanum lycopersicum (tomato) from neighbor joining phylogenies based on their protein kinase domain from Prosite (https://prosite.expasy.org). (DOCX 32 kb) [file 12870_2019_1804_MOESM1_ESM.docx]

**Additional file 1**

**Table S1: List of primers**

| **Name of the primer** | **Sequence** | **Size of amplicon** | **Number of cycles** |
| --- | --- | --- | --- |
| FRK1 RT-PCR F | 5’-CCAAAGAAGAGGAAGAGGCAAC-3’ | 343 | 28 |
| FRK1 RT-PCR R | 5’-GGTGAGAAATAACCCATACGACG-3’ |  |  |
| FRK2 RT-PCR F | 5’-GGATTCGCAAGCAAGGTTGG-3’ | 379 | 28 |
| FRK2 RT-PCR R | 5’-CCTTCCTCTTCCTCTGATGAAC-3’ |  |  |
| FRK3 RT-PCR F | 5’-GTCCATTTGATTTCCCAGATTG-3’ | 343 | 28 |
| FRK3 RT-PCR R | 5’-GTGGCAACCAATGTTATGTAC-3’ |  |  |
| FRK4 RT-PCR F | 5’-GCAACAGTGGCAGCCCTTCAAC-3’ | 338 | 28 |
| FRK4 RT-PCR R | 5’-TGGCCTAGCCTAATCACTAAATTG-3’ |  |  |
| FRK5 RT-PCR F | 5’-GAAATCGCCTCTTCGTTTGCTG-3’ | 227 | 28 |
| FRK5 RT-PCR R | 5’-CTACAGAGAGTGTAGCCAAATAAG-3’ |  |  |
| FRK6 RT-PCR F | 5’-GCAATTGCTAGATCCGAAGC-3’ | 288 | 28 |
| FRK6 RT-PCR R | 5’-GGATCAAACCCATACATACTTC-3’ |  |  |
| Actine RT-PCR F | 5’-TGAGTTACCAGATGGTCAGG-3’ | 228 | 23 |
| Actine RT-PCR R | 5’-TCCTTGCTCATACGATCAGC-3’ |  |  |
| ScMKK3 RT-PCR F | 5’-GAGGGTCTGCCAAACTTGAAAG-3’ | 198 | 28 |
| ScMKK3 RT-PCR R | 5’-CCATTTACAGGAACACACACAAC-3’ |  |  |
| ScMPK13 RT-PCR F | 5’-GTTGAGGATGCATTGAACCATCC-3’ | 232 | 28 |
| ScMPK13 RT-PCR R | 5’-GCTAAACCTGCCACAACCAAATC-3’ |  |  |
| FRK3 ORF F | 5’-ATGGATTGGGTTCGTGGTG-3’ | 1059 |  |
| FRK3 ORF R | 5’-TCACCTAACAGTAACCCAG-3’ |  |  |
| FRK3 in situ T3 F | 5’-GCAATTAACCCTCACTAAAGGGCAAAGAGAGTTGATGATG-3’ | 603 |  |
| FRK3 in situ T7 R | 5’-TAATACGACTCACTATAGGGTCACCTAACAGTAACCCAG-3’ |  |  |
| FRK3 Npart F | 5’-GGGGCCATGGAGGATTGGGTTCGTGGTGAAGC-3’ |  |  |
| FRK3 Npart R | 5’-GGGGGGATCCGTGACCATCTCCGGCGACATG-3’ |  |  |
| FRK3 Cpart F | 5’-GGGGCCATGGAGCGGCGGCGAACAGGGTAC-3’ |  |  |
| FRK3 Cpart R | 5’-GGGGGGATCCTCACCTAACAGTAACCCAGTCA-3’ |  |  |
| FRK3 Midpart F | 5’-GGGGCCATGGAGGAGTATGCTACTGGAGGTTCG-3’ |  |  |
| FRK3 Midpart R | 5’-GGGGGGATCCCGTCCATCTCTTTCTTGGATC-3’ |  |  |

**Table S2**: MKKs and MPKs found in *Solanum chacoense* ovule and pollen transcriptomes and their closest orthologs in *Arabidopsis thaliana* and *Solanum lycopersicum* (tomato) from neighbor joining phylogenies based on their protein kinase domain from Prosite (<https://prosite.expasy.org>).

|  | *S. chacoense* transcript | Closest *A. thaliana* ortholog | Closest *S. lycopersicum* ortholog | Group |
| --- | --- | --- | --- | --- |
| MKKs | isotig13756 | AtMKK1, 2 | SlMKK1 | A |
|  | isotig15708 | AtMKK3 | SlMKK5 | B |
|  | isotig25857 | AtMKK7, 8, 9 | SlMKK4 | D |
|  | isotig37602 | AtMKK4, 5 | SlMKK2 | C |
|  | isotig38529 | AtMKK6 | SlMKK3 | A |
| MPKs | isotig07712 | AtMPK18, 19 | SlMPK10 | D |
|  | isotig10718 | AtMPK8, 9, 15 | SlMPK16 | D |
|  | isotig16044 | AtMPK4, 11 | SlMPK6 | B |
|  | isotig17837 | AtMPK16 | SlMPK13 | D |
|  | isotig27038 | AtMPK17 | SlMPK14 | D |
|  | isotig27937 | AtMPK1, 2, 7, 14 | SlMPK8 | C |
|  | isotig34221 | AtMPK20 | SlMPK12 | D |
|  | isotig34270 | AtMPK18, 19 | SlMPK11 | D |
|  | isotig34923 | AtMPK8, 9, 15 | SlMPK16 | D |
|  | isotig35805 | AtMPK1, 2 | SlMPK9 | C |
|  | isotig36077 | AtMPK4, 11 | SlMPK5 | B |
|  | isotig36598 | AtMPK12 | SlMPK7 | B |
|  | isotig37161 | AtMPK6 | SlMPK1 | A |
|  | isotig37197 | AtMPK3 | SlMPK3 | A |
|  | isotig37281 | AtMPK6 | SlMPK2 | A |
|  | isotig37333 | AtMPK13 | SlMPK4 | B |

**ScMKKs (5)**

>isotig37602

MRPTQPAANSTSSAAASSMPPPSSAGQRRRPRRRTDLTLPLPQRDVALAVPLPLPPTSAPSSSSSSSSSPLPTPLHFSELERVNRIGSGTGGTVYKVLHRPTGRLYALKVIYGNHEDSVRLQMCREIEILRDVDNPNVVRCHGMFDHNGEIQVLLEFMDKGSLEGIHIPLEQPLSDLTRQVLSGLYYLHRRKIVHRDIKPSNLLINSRREVKIADFGVSRVLAQTMDPCNSSVGTIAYMSPERINTDLNHGQYDGYAGDIWSLGVSILEFYLGRFPFSVGRQGDWASLMCAICMSQPPEAPPTASREFREFIACCLQRDPARRWTAAQLLRHPFITQNSPGTHTGPATTSLSNQAHQLLPPPPHFSSSSSSS

>isotig25857

MALVRDRRHLNLRLPLPEPSERRPRFPLPLPPSSVSTVNSTANTTTTTTTTTTASTTTISISELEKLKVLGHGNGGTVYEVRHKRTSAIYALKVVHGDSDPEIRRQILREISILRRTDSPYVIKCHGVIDMPGGDIGILMEYMNAGTLENLLKSQSTFSELCLAKIAKQVLGGLDYLHSHKIIHRDLKPSNLLVNREMEVKIADFGVSKIMGRTLDPCNSYVGTCAYMSPERFDPDTYGGNYNGYAADIWSLGLTLMELYMGHFPFLPPGQRPDWATLMCAICFGEPPSLPENTSEKFNDFMKCCLQKESSKRWSAHQLLQHPFIQSIDLKST

>isotig13756

MKKGSFAPNLKLSLPPPDEVALSKFLTESGTFKDGDLLVNRDGVRIVSQSEVAAPSVIQPSDNQLCLADFEAVKVIGKGNGGIVRLVQHKWTGQFFALKVIQMNIDESMRKHIAQELRINQSSQCPYVVICYQSFFDNGAISLILEYMDGGSLADFLKKVETIPERYLAVICKQVLKGLWYLHHEKHIIHRDLKPSNLLINHRGDVKITDFGVSAVLASTSGLANTFVGTYNYMSPERISGGAYDYKSDIWSLGLVLLECATGHFPYTPPEGDEGWVNVYELMETIVDQPEPCAPPDQFSPQFCSFISACVQKHQKDRLSANELMSHPFITMYDDQDIDLGSYFTSAGPPLATLTEL

>isotig38529

MKTAKPLKQLKLSVPAQDTPISSFLTASGTFHDGDLLLNQKGLRLISEENESLPSETKEIDLQFSLEDLETIKVIGKGSGGVVQLVRHKWVGTLFALKVIQMNIQEDIRKQIVQELKINQASQCPHVVVCYHSFYHNGAISLVLEYMDRGSLVDVIGQLKTILEPYLAVVCKQVLQGLVYLHHERHVIHRDIKPSNLLVNHKGEVKITDFGVSAMLASSMGQRDTFVGTYNYMAPERISGSTYDYKSDIWSLGMVILECAIGRFPYIQSEDQQARPSFYELLEAIVSSPPPSAPADQFSPEFCSFVSACIQKDPRDRSSALDLLSHPFIKKFEDKDIDLSILVSSLEPPVNFPR

>isotig15708

MAGLEELKKKLVPLFDADKGFSPTSTSDPFDSYSLSDAGTVNLLSQSYGVYNINELGLQKWPVDDTDHGEKTYRCASHEMRVFGAIGAGASSVVQRAIHIPTHRIIALKKINIFEKEKRQQLLTEIRTLCEAPCYQGLVEFYGAFYTPDSGQISIALEYMDGGSLADIIKIRKSIPEPILSSMVQKLLHGLSYLHGVRHLVHRDIKPANLLVNLKGEPKITDFGISAGLESSIAMCATFVGTVTYMSPERIRNENYSYPADIWSLGLALFECGTGEFPYTANDGPVNLMLQILDDPSPSLSRHDYSPEFCSFVDACLKKNPDDRPTADQLLSHPFTIKYSDSALDLGTFVRDIFDPTQRMKDLADMLTIHYYLLFDGSDEFWQHTKTLYNECSTFSFGGKESIGPNNIFSTLSNIRNTLAGEWPPEKLVHVVEKLQCRANGQDGVAIRVSGSFIVGNQFLICGDGMQVEGLPNLKDLSIDIPSKRMGTFHEQFIVEKANIIGRYFITKHELFITQ

**ScMPKs (16)**

>isotig35805

MATPVEPPNGIRSPGKHYYSMWQSLFEIDTKYVPIKPIGRGAYGIVCSSVNRETNEKVAIKKINNAFENRVDALRTLRELKLLRHLRHENVIALKDVMMPIHRPSFKDVYLVYELMDTDLHQIVKSSQTLTNDHCQYFLFQLLRGLKYLHSANILHRDLKPGNLLINANCDLKICDFGLARTSSGKDQFMTEYVVTRWYRAPELLLCCDNYGTSIDVWSVGCIFAELLGRKPVFPGTECLNQLKLIINILGSQREEDLEFIDNPKARKYIKSLPYSPGTPFSRLYPQAHPLAIDLLQRMLVFDPSKRISVMEALQHPYMSPLYDPNTDPPAQVPINLDIDEDLGEETIRDMMWTEILHYHPEAATAAMEEVM

>isotig27937

MATQVEAPNGIRSRGKHYYTMWQTVFEVDTKYVPIKPIGRGAYGVVCSSVNRETNEKVAIKKINNVFSNRIDALRTLRELKLLRHIRHENVIALKDVMMPIHRNSFKDIYLVYELMDTDLNHIIKSPQPLSDDHCKYFLFQLLRGLKYLHSANILHRDLKPGNLLVNANCELKICDFGLARTSRDNGQFMTEYVVTRWYRAPELLLCCDNYGTSIDVWSVGCIFAEILGRKPLFPGTECLNQLKLILNILGSQPEADLHFIDNPRAKGFIRSLPFTRGAHFSSLFPNADPLAIDLLQRMLIFDPSKRITVTEALYHPYLSSLFDPTCNLPAQFPLNLNIDENMAEPLIREMMLREIFHYHPEAAYINTFY

>isotig37197

MVDANMSGAQFPDFPKIVTHGGQYVQYDIFGNYFEITNKYRPPIMPIGRGAYGIVCSVFNAELNEMVAVKKIANAFDNYMDAKRTLREIKLLRHLDHENVIGLRDVIPPPLRREFSDVYIATELMDTDLHQIIRSNQGLSEDHCQYFMYQLLRGLKYIHSAHVIHRDLKPSNLLLNANCDLKICDFGLARPNVENENMTEYVVTRWYRAPELLLNSSDYTAAIDVWSVGCIFMELMNRKPLFAGKDHVHQIRLLTELLGTPTESDLSFLRNEDAKRYVRQLPQHPRQQLATVFPHVNPLAIDLVDKMLTLDPTRRITVEDALAHPYLAKLHDAADEPVCPVPFSFDFEQQGIGEEQIKDMIYQEALALNPEYA

>isotig37161

MDVSAPQTDTMMPDVAAPAVQQPPPPPQPLPGMDNIPATLSHGGRFIQYNIFGNIFEVTAKYKPPIMPIGKGAYGIVCSALNSETNENVAIKKIANAFDNKIDAKRTLREIKLLRHMDHENIVAIRDIIPPPQREAFNDVYIAYELMDTDLHQIIRSNQGLSEEHCQYFLYQILRGLKYIHSANVLHRDLKPSNLLLNANCDLKICDFGLARVTSETDFMTEYVVTRWYRPPELLLNSSDYTAAIDVWSVGCIFMELMDRKPLFPGRDHVHQLRLIMELIGTPSEAEMEFLNENAKRYIRQLPLYRRQSFTEKFPHVNPAAIDLVEKMLTFDPRRRITVEDALAHPYLTSLHDISDEPICMTPFNFDFEQHALTEEQMKELIYRESIAFNPEYQRM

>isotig37281

MDGSAPQTDTVMSDAAAGQQPATPPLPMAGMENIPATLSHGGRFIQYNIFGNIFEVTAKYKPPIMPIGKGAYGIVCSALNSETNEHVAIKKIANAFDNKIDAKRTLREIKLLRHMDHENIVAIRDIIPPPQREAFNDVYIAYELMDTDLHQIIRSNQGLSEEHCQYFLYQILRGLKYIHSANVLHRDLKPSNLLLNANCDLKICDFGLARVTSETDFMTEYVVTRWYRPPELLLNSSDYTAAIDVWSVGCIFMELMDRKPLFPGRDHVHQLRLLMELIGTPSEAEMEFLNENAKRYIRQLPLYRRQSFVEKFPHVNPAAIDLVEKMLTFDPRRRLTVEGALAHPYLTSLHDISDEPVCMTPFSFDFEQHALTEEQMKELIYREGLAFNPEYQHM

>isotig37333

MDAENIENSVEIKGIPTRDGKYVEYNVVGNLFEVTSKYVPPIQPVGRGAYGIVCCATNSETKEEIAIKKIGNAFENRIDAKRTLREIKLLSHMDHENVIKIKDIVRPPDREEFNDVYIVYELMDTDLHQIIRSSQALTEDHCQYFLYQLLRGLKYVHSANVLHRDLKPSNLLLNANCDLKICDFGLARTTSEADFMTEYVVTRWYRAPELLLNCTEYTAAIDIWSVGCILMELIKREPLFPGRDYAQQLGLIIKLLGSPEESDLGFLRSDNARKYVKQLPQVPKQPFSEHFPDVSPLALDLAEKMLVFDPAKRITVEDALNHPFMISLHEINEEPVCTSPFNFDFEQASLSEEDIKELIWNEALKFDPDTTK

>isotig16044

MSLDSSSADHGGHSNIRGIPTHGGRYVQYNVHGSLFEVSRKYVPPIRPIGRGANGMVCAAVNSETREEVAIKKIGNAFDNVIDAKRTLREIKLLSHMDHENVIAIKDVIRPPQKKNFNDVYIVYELMDTDLHQIIHSSQQLTDEHCRHFLYQILRGLKYIHSANILHRDLKPSNLLVNAKCDLKIGDFGLARTTTETDFMMEYCVTRWYRAPELLLNCSEYTSAIDIWSVGCILGEILTRQPLFPGRDYVHQLRLITELIGSPDDASLGFLRSNNARRYVRQLPRYPRQQFAARFPNSSPRAVDLLEKMLIFDPSRRITADEALCHPYLAPLHEINEEPVCPRPFSLDFEQPSLTEDNIKELIWREAAKFNPDPTH

>isotig36077

MEASPGDHGVQSNFNGVPTHGGRYVQYNVYGNLFEVSKKYVPLRPVGRGAYGIVCAALNSETREEIAIKKIGNAFDNRIDAKRTLREIKLLRHMDHDNVIAMKDIIRPPQTENFNDVYIVYELMDTDLHQIIRSNQQLTDDHCRYFLYQILRGLKYIHSANVLHRDLKPSNLLLNANCDLKVGDFGLARTTSETDFMTEYVVTRWYRAPELLLNCSEYTAAIDIWSVGCILGEMMTRQPLFPGRDYVHQLKLITELIGSPDDASLGFLRSDNARRYVRQLPQYPRQQFAAKFPNSSPGAVDLLEKMLVFDPSRRVTVDEALCHPYLAPLHDINEEPVCPMPFSFDFEQPSFTEENIKELIWRESVNFNPDPTH

>isotig36598

MANQTGGASSTGSRDIRGVLIHGGQYVRYNVYGSLFEVSSKYVPPIRPIGRGAYGLVCAAVNSETREEVAIKKIGNAFDNRIDAKRTLREIKLLRHLDHENIVAIKDLIRPPKKEAFNDVYIVSELMDTDLHQIIRSEQPLTNDHCQYFMYQLLRGLKYVHSANVLHRDLKPSNLFLNANCDLKIGDFGLARTTSETDFMTEYVVTRWYRAPELLLNCSEYTGAIDVWSVGCIFGEIMTREPLFPGKDYVQQLRLITELLGSPDDASLQFLRSDNARRYVRQLPQYPKQQFSARFPSMSPLAVDLLEKMLVFDPTRRITVDEALCHPFLSSLHDLNDEPICPRPFSVDFDEPSITEEKIKELIWRESVKFYPDFVEQNI

>isotig34923

MGGSSSFVDGVLRWFHRRHTNEDAILTQPHNSADTHLQEKQENKHEFTITEDFDFTGLKLIKVPKRFHLPISSSSMDPIKKNALETEFFTEYGEASRYQVQEVIGKGSYGVVGSAVDTHTGERVAIKKINDVFDHVSDATRILREIKLLRLLRHPDIVEIKHIMLPPSRREFKDIYVVFELMESDLHQVIKANNDLTHEHYQFFLYQLLRGLKYIHTANIFHRDLKPKNILANADCKLKICDFGLARVSFNDVPSAIFWTDYVATRWYRAPELCGSFFSKYTPAIDIWSIGCIFAELLSGKPLFPGKNVVHQLDLITDLLGTPPPETVAKIRNEKARRYLSSMRKKQPVGFAKKFPNADPLALRLLERLLAFDPKDRPSAEEGLEDPYFHGLSNADREPCRPPISKLEFEFEKRKLAKDDVRELIYREILEYHPQMLQEYLSGGDQTSGFMYPSGVDRFKRQFAHLEEHAGKGEHSAPILRQHASLPRERVPAPKDDTSSQNNDCEKRTVSTTLQSPPAQSEGSENYSARSLLKSASISGSKCVEVKRRNAEEEPIEEQNEEVDGLSQEVASLHV

>isotig10718

MGGGSTFVDGVRRLFHRRSSTTTTITTIVHTNAEPILNNLEKPIKISDSQYQQEEEGELIIIEDFDISGLKFIRVPKRVDFPFSSASYQMDHHRKNSQEAEFFTEYGEASRYQIQEVIGKGSYGVVASAVDTKTGERVAIKKINDVFEHVSDATRILREIKLLRLLRHPDIVEIKHIMLPPSRREFKDIYVVFELMESDLHQVIKANDDLTPEHYQFFLYQLMRGLKYTHTANVFHRDLKPKNILANADCKLKICDFGLARVSFNDTPSAIFWTDYVATRWYRAPELCGSFFSKYTPAIDIWSIGCIFAEMLTGKPLFPGKNVVHQLDLMTDLLGTPPPETIAKIRNEKARRYLGNMRKKPPVPYAQKFPHADPLALRLLERILAFDPKDRPSAEEALADPYFSSLSNVDREPSTHPISRLEFEFERRKLAKEDVRGLIYREILEYHPQMLQEYHRGGDQTSGFMYPSSVDRFKRQFAHLEEHYGKGERSTPLQRQHASLPRERVPAPKDETSSQNIDFEKQTSASVASTLESPPGQSEGSEYSGANVQNGPNKANYSARSLLKSASISGSKCVVVKRRNAEEEAIKEQTDGVDGLAQKLADLHG

>isotig27038

MVMDFKEFFTEYGEAHQYEIQEVVGKGSYGVVAAAIDTHTGEKVAIKKINDVFEHSCEATRILREIKLLRLLRHPDIVEIKHILLPPCPREFKDIYVVFELMECDLQHVIKANDSLTAEHYQFFMYQLLRGLKYMHTANVFHRDLKPKNILANADCKLKICDFGLARVSLGDNPSAVFWTDYVATRWYRAPELCGSFFSKYTPAVDIWSLGCIFAEMLTGKPLFPGKNAVHQLDLITDLLGTPSTEAISRIKNEKARRYLSSMKKKAPIPLSQQFPHVDPLALRLLERLIAFDPKDRPSAEEALAHPYFRGLANKEQEPSSQTISKFEFDFERRKLGKEDIRELIYREILEYHPQMLQEHLHGTDHTHFMYPSGVDCFKQQFDHLEGHSGRGGSTLFPRRYASLPRERVCASTDEEADKDGEFERHVVASMAQRCLPSPPSSPKVKKPDTANTVEGPTEAGHSTPMNTERCMLRSSSISFSKCVGAIWDCEVSVF

>isotig17837

MNYNMNMQGSGDVDFFTEYGEGSRYKIEEVIGKGSYGVVCSAYDTHLGEKVAIKKINDIFEHVSDATRILREIKLLRLLRHPDIVEIKHILLPPSRREFKDIYVVFELMESDLHQVIKANDDLTPEHYQFFLYQLLRGLKYIHTANVFHRDLKPKNILANADCKLKICDFGLARVAFNDTPTAIFWTDYVATRWYRAPELCGSFFSKYTPAIDIWSIGCIFAELLTGKPLFPGKNVVHQLDLMTDLLGTPSPESIARIRNEKARRYLSSMRKKKSVPFSHKFPHADPLALRLLERMLAFDPKDRPNAEEALADPYFRNLAKVEREPSAQPVTKMEFEFERRRITKEDVRELIYREILEYHPKMLKEFLEGQEPTSFMYPSAVDKFKKQFAYLEEHYGKGGAAAPPERQHSSSLPRACVLYSDNSVQNPVEVANDLSKCSIKEDEKPQADRSSMIPMTRLPLQVPQNVQGGAARPGRVVSSVLRYNNCGAAATAAEVIEQRRIARNPGGPTQYPISNTSYPRRHPSCKNERGEDSTEVSNGVQPKPEQYIARKVAAAPGGPGNQWY

>isotig07712

MMQQDHRKKNTKEVEFFTEYGEANRYKILEVVGKGSYGVVCAAIDTHTGEKVAIKKITDIFEHASDAIRILREIKLLRLLRHPDIVDIKRIMLPPSKRDFKDIYVVFELMESDLHHVIKANDDLSHEHHRFFLYQMLRALKYMHTANVYHRDLKPKNILANANCKLKICDLGLARVSFNDTPTTALWTDYVATRWYRAPELCGSFFSKYTPAIDIWSIGCIFAEVLTGKPLFPGKSVVHQLDLMTDLLGTPSADTISGVRNEKARKYLTSMRKKNPVPFTEKFPGADPLVLRLLQRLLAFDPKDRPTAEEALSDPYFKGLAKIEREPSCRPISNLEFEFEKRRLTKEDIKELIFREILEYHPQLQKDYIAGNDGTNYLYPSATGQFRRQFAYLEENNGKSGTVIPLGRKHVSLPRSAVNSSTDPPKARQNFSVFDHTQVTEKSSTDVRVAEKISGTIQNISRPPHRVHAAKPGRVVGPILPYDGRMLTQNTGLLPHGISPHYMFRMNPGNREKCGTEAKDTTQVRPLPAQCNMGVEMNTNPYYPTQEKVVQLGGQIAIDAKLLQAQTQFGAVSAAAVAVAAHREVGTIQYSLT

>isotig34270

MQQDQHKKSSKEVEFFTEYGDANRYKILEVVGKGSYGVVCAAIDTHTGEKVAIKKITDIFEHISDAIRILREVKLLRLLRHPDIVEIKRIILPPSRREFRDIYVVFELMESDLHHVIKANDDLTQEHHRFFLYQMLRALKFMHTANVYHRDLKPKNILANANCKLKICDFGLARVAFSDTPTTTFWTDYVATRWYRAPELCGSFFSKYTPAIDIWSIGCIFAEVLTGKPLFPGKSVVHQLDLITDLLGTPSVDIVSGVRNEKARKYLTDMKKKSPVPFTEKFRKADPLALKLLQRLLAFDPKDRPTAEEALADPYFKGLAKIEREPSSQPISKLEFEFERRRVTKDDIRELIFREILEYHPQLLKDYMAGNSGANFLYPSAIGNFRRQFAYLEENSGKSGPVIPPGRKHVSLPRSTVNSSTIPPRTQQNPMFDHRQVTEKATAGIRVSDPKVLRPPPRVPTAKPGRVLGPVLSYDGDRSIKVTDGRVYPQNSVVQPHGMSPHYLFRSNSTHLERCGTEAEKDRSQVKLQHGQCMVAKSSASMSFEMNTNPYYHTQARVAQLGGQIAMDAKLLQAQTQFGAVGAAAVAVAAHREVGTVQYGLT

>isotig34221

MQPDHRKKSSAEMDFFSEYGDANRYKIQEVIGKGSYGVVCSAIDTHTGEKVAIKKIHDIFEHISDAARILREIKLLRLLRHPDIVEIKHIMLPPSRRDFKDIYVVFELMESDLHQVIKANDDLTREHYQFFLYQLLRALKYIHTANVYHRDLKPKNILANANCKLKICDFGLARVAFNDTPTTIFWTDYVATRWYRAPELCGSFYSKYTPAIDIWSIGCIFAEVLTGKPLFPGKNVVHQLDLMTDLLGTPSMDTISRVRNDKARRYLTSMRKKQPVSFAQKFPNADPLSLKLLERLLAFDPKDRPTAEEALADPYFKGLAKSEREPSCKSISKMEFEFERRRVTKEDLRELIFREILEYHPQLRKDYLNGVERTNFLYPSAVDQFRKQFAHLEENGANGVPVVPMDRKHVSLPRSTVVHSNPNPLKEQPIAANMRDRQNGEESCSRNCRDSEGLASSLTRTLQAQPRNALAAKPGKVVGPGLAYDSGNREKYDPRPQVRNAVGPPQIMSSVYNYDRSGVVKQERSVETERGMNSHSKPMAPCGMAAKLSPDIAINIDSNPFYMMRAGVTKPDRVDDRITIDTNLLQAKSQYGGIGVAAAAATTGAAHRKVGTVQYGMSRMY
