# Supplementary material for: The Solanum chacoense Fertilization-Related Kinase 3 (ScFRK3) is involved in male and female gametophyte development
Source: BMC Plant Biol. 2019 May 16;19:202. doi: 10.1186/s12870-019-1804-0 (PMC6524262; doi:10.1186/s12870-019-1804-0)
Supplement: Supplementary file 2 — Figure S1. Phylogenetic tree showing the relations between the AtMKKs, the SlMKKs and the ScMKKs found in the S. chacoense ovule and pollen transcriptome. In tomato, the closest ortholog of the S.chacoense isotig15708 (ScMKK3) is Solyc03g019850, named SlMKK5 (ITAG release 3.2; http://solgenomics.net) with 99% amino acid sequence identity. The MKKs are classified in four groups, from A to D. The tree was made by the Neighbor Joining method using 1000 replicates and rooted with the Arabidopsis BRI1 receptor kinase using MacVector 17 software. Only the kinase domain was used for the alignment. (PDF 137 kb) [file 12870_2019_1804_MOESM2_ESM.pdf]

# Figure S1

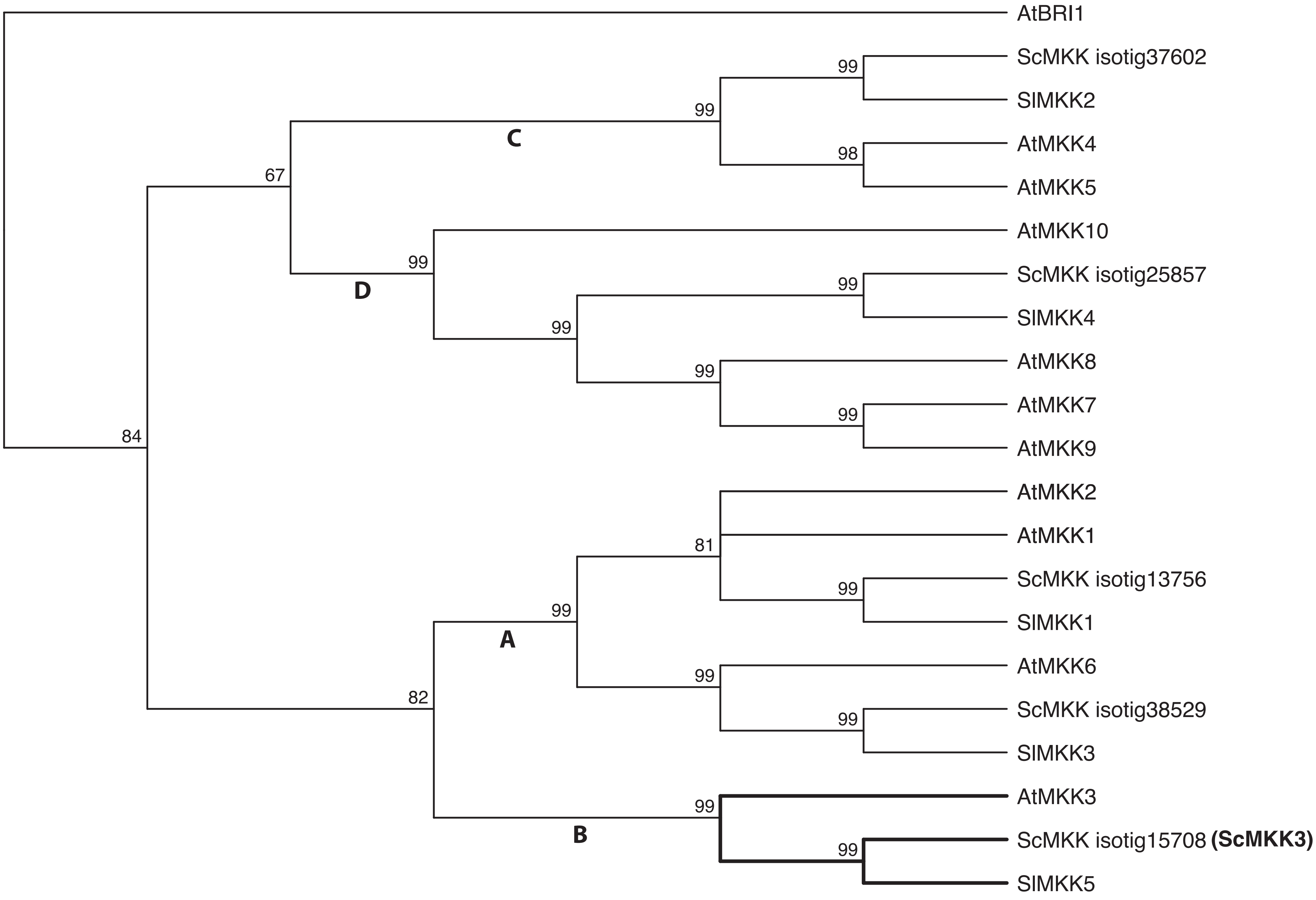


Method: Neighbor Joining; Bootstrap (1000 reps); tie breaking = Random
Distance: Poisson-correction
Gaps distributed proportionally
