# Supplementary material for: The Solanum chacoense Fertilization-Related Kinase 3 (ScFRK3) is involved in male and female gametophyte development
Source: BMC Plant Biol. 2019 May 16;19:202. doi: 10.1186/s12870-019-1804-0 (PMC6524262; doi:10.1186/s12870-019-1804-0)
Supplement: Supplementary file 3 — Figure S2. Phylogenetic tree showing the relations between the AtMPKs, SlMPKs and the ScMPKs found in the S. chacoense transcriptome. The MPKs are classified in four groups, from A to D. The tree was made by the Neighbor Joining method using 1000 replicates and rooted with the Arabidopsis BRI1 receptor kinase using MacVector 17 software. Only the kinase domain was used for the alignment. (PDF 155 kb) [file 12870_2019_1804_MOESM3_ESM.pdf]

Figure S2

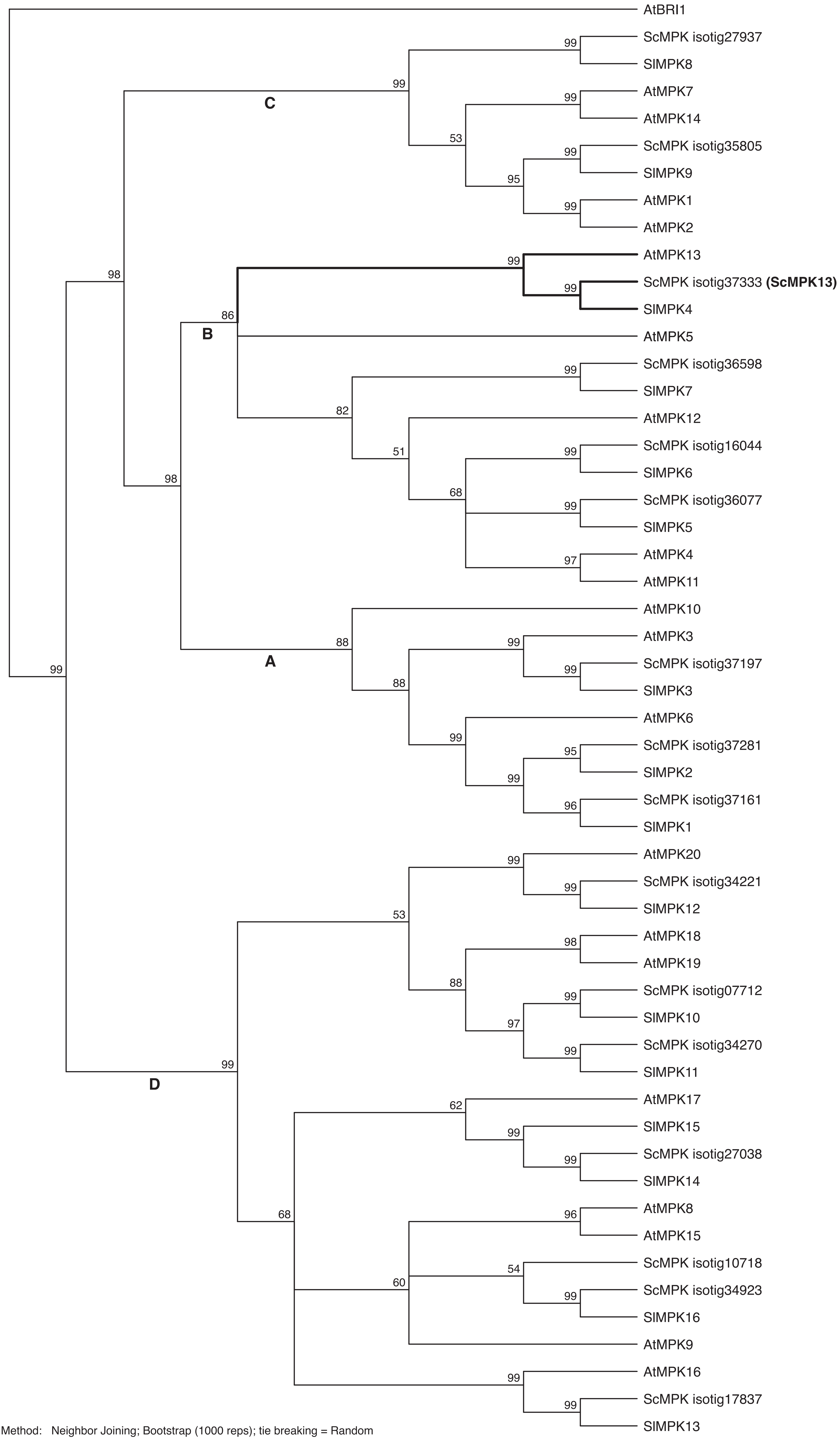

Method: Neighbor Joining; Bootstrap (1000 reps); tie breaking = Random
Distance: Poisson-correction
Gaps distributed proportionally
