# Supplementary figures and images for: The Solanum chacoense Fertilization-Related Kinase 3 (ScFRK3) is involved in male and female gametophyte development
Source: BMC Plant Biol. 2019 May 16;19:202. doi: 10.1186/s12870-019-1804-0 (PMC6524262; doi:10.1186/s12870-019-1804-0)

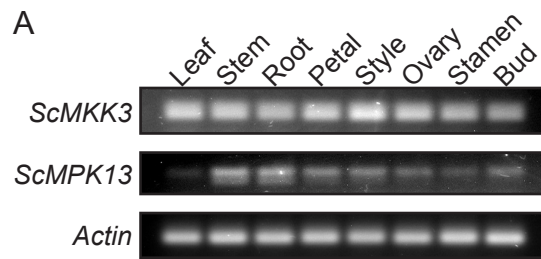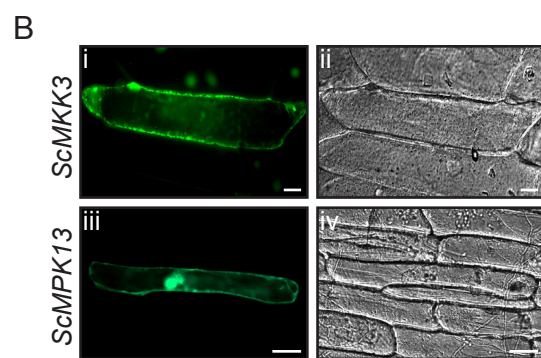

Supplement: Supplementary file 4 — Figure S3. ScMKK3 and ScMPK13 expression and localization. A. Expression profiling on different tissues for ScMKK3 and ScMPK13 using semi-quantitative RT-PCRs. B. Protein localization of ScMMK3 and ScMPK13 using microparticle bombardment. ScMKK3-GFP localization in onion cell (i) and DIC image. ScMPK13-GFP localization in onion cell (iii) and DIC image (iv). Scale bars: 50 μm. (PDF 236 kb) [file 12870_2019_1804_MOESM4_ESM.pdf]
