# Supplementary material for: The Solanum chacoense Fertilization-Related Kinase 3 (ScFRK3) is involved in male and female gametophyte development
Source: BMC Plant Biol. 2019 May 16;19:202. doi: 10.1186/s12870-019-1804-0 (PMC6524262; doi:10.1186/s12870-019-1804-0)
Supplement: Supplementary file 5 — Figure S4. Conservation of the C-terminal ScFRK3 segment and its closest genes in S. chacoense and A. thaliana. The AtMKKK20 C-terminal segment (285–342) was shown to interact with AtMKK3 and harbored a typical DEF mammalian MAP kinase docking site (Docking site for ERK, FXFP) identical to the S. chacoense ScFRK3 and 4, as well as the two closest MKKKs in A. thaliana [48]. DEF domains are generally characterized by a FXF [P/D/E)] motif located between 6 and 20 amino acids C-terminal to the [S/T]-P phosphoacceptor site [49]. (PDF 269 kb) [file 12870_2019_1804_MOESM5_ESM.pdf]

```
ScFRK3    261 ADHDTVTLLNEEI-KNGVPSISPKCPFDFPDWVSDESAQSSVTCSITFLPSPE---NLNSSCGRWSTSPAERLMGLVSEFSTESEWCSHDDWVTVR 352
ScFRK4    265 ADEDDTVLLNEERCNSGSPSTSPRCPFDFPDWVSNKSAESSVTCSITSLPSPAFQESMNWSDESWSTSPTERIRELVCECRPEFEWSTADGWVSVR 360
AtMKKK19  289 KTED--------------VSTSPRCPFEFPDWVSVSSG-S-----------------------QTIDSPDERVASLVTDMIP--DWSVTNSWVTVR 344
AtMKKK20  285 KDEDK-------------VLMSPKCPFEFDDWDSFTLDSN-----------------------PSFDSPVERLGSLVSGSIP--DWSVGGSWLTVR 342
AtMKKK21  273 EEDEA-------------CSVSPRNPFDFPGWNSVQSPVN-----------------DSVMFGSLVGSPEERISGLVSEKVP--DWSVSCDWVNVR 336
                                   SP   FxF                                      SP
```
